# Challenges and strategies in developing high-performance n-type polycrystalline SnSe thermoelectric materials

**DOI:** 10.1016/j.isci.2025.114025

**Published:** 2025-11-14

**Authors:** Adeel Abbas, Habib Khan, Ch Asad Abbas, Syed Irfan, Hamedalneel B.A. Hamid, Jamal N.A. Hassan, Farhan Mudasar, Tianjun Ma, Yue-Xing Chen, Muhammad Shahid Rafique

**Affiliations:** 1 General Education Centre, Quanzhou University of Information Engineering, Quanzhou, Fujian 362000, China; 2 School of Electronics and Communication Engineering, Quanzhou University of Information Engineering, Fujian 362000, China; 3 Guangdong Provincial Key Laboratory of Micro/Nano Optomechatronics Engineering, College of Mechatronics and Control Engineering, Shenzhen University, Shenzhen 518060, China; 4 State Key Laboratory of Environment-friendly Energy Materials, Southwest University of Science and Technology, Mianyang 621010, P.R. China; 5 Department of Physical and Environmental Sciences, University of Toronto Scarborough, Toronto, ON, Canada; 6 Zhongshan Institute of Advanced Cryogenic Technology, Zhongshan, China; 7 Guangdong Green Peak Energy Technology Corporation Limited, Zhongshan, China; 8 Shenzhen Key Laboratory of Advanced Thin Films and Applications, Key Laboratory of Optoelectronic Devices and Systems of Ministry of Education and Guangdong Province, College of Physics and Optoelectronic Engineering, Shenzhen University, Shenzhen 518060, China; 9 Laser and Optronics Centre, Department of Physics, University of Engineering and Technology, Lahore, Pakistan

**Keywords:** Chemistry, Physics, Engineering, Materials science

## Abstract

This review comprehensively examines recent advances in n-type SnSe thermoelectric materials, addressing the critical performance gap with their p-type counterparts. We analyze the fundamental challenges imposed by intrinsic defect chemistry, anisotropic transport, and doping limitations that have historically constrained n-type SnSe development. The discussion systematically evaluates synthesis approaches spanning bulk crystal growth to nanostructured thin films, highlighting how processing conditions influence microstructural evolution and thermoelectric properties. A central focus is placed on doping strategies, including halogen-based and transition metal systems that enable carrier concentration optimization and phonon scattering enhancement. By synthesizing theoretical insights with experimental breakthroughs, this review identifies key knowledge gaps and proposes actionable research directions to realize high-performance n-type SnSe materials, ultimately enabling the development of efficient SnSe thermoelectric devices.

## Graphical abstract

**Figure:**
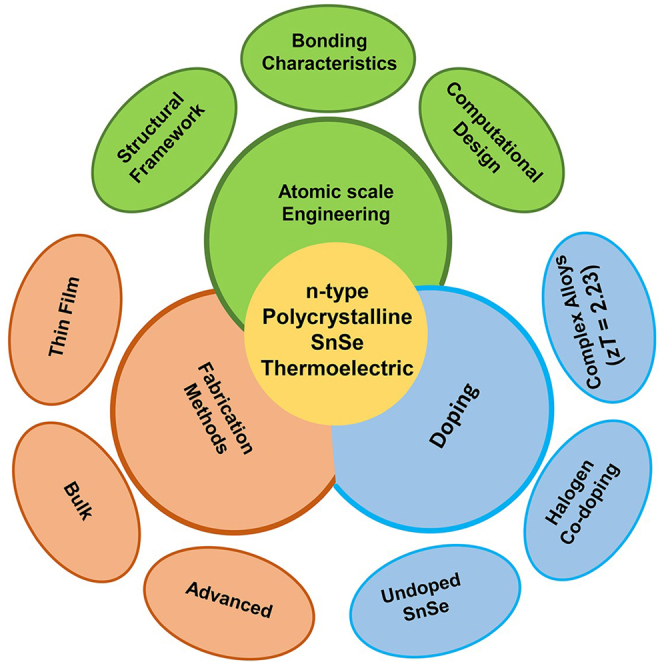

## Introduction

The thermoelectric effect, with its dual manifestations in the Seebeck (1821) and Peltier (1834) phenomena, represents one of the most elegant energy conversion mechanisms, establishing the foundation for modern thermoelectric materials capable of direct heat to electricity conversion for power generation or refrigeration.^1^^,^^2^^,^^3^^,^^4^^,^^5^ Over nearly two centuries of development, thermoelectric materials have evolved from scientific curiosities to technologically viable solutions for sustainable energy applications, offering unique advantages such as silent operation, zero greenhouse gas emissions, and exceptional reliability due to the absence of moving parts.^6^^,^^7^^,^^8^ At the heart of this technology lies the dimensionless figure of merit (*ZT* = *S*^2^*σT*/*κ*), which quantifies conversion efficiency through three interdependent parameters: the Seebeck coefficient (*S*) measuring induced voltage per temperature gradient, electrical conductivity (*σ*) reflecting charge carrier mobility, and total thermal conductivity (*κ* = *κ*_*ele*_+*κ*_*latt*_) encompassing both electronic and lattice contributions to heat transport.^9^^,^^10^^,^^11^^,^^12^^,^^13^^,^^14^^,^^15^^,^^16^ This fundamental relationship encapsulates the central challenge of thermoelectric material design, which is the need to simultaneously maximize power factor (*PF* = *S*^2^*σ*) while minimizing *κ*, objectives that are intrinsically opposed due to their coupling through the Wiedemann Franz law, which dictates that enhancements in electronic transport typically increase electronic thermal conductivity proportionally.^17^^,^^18^^,^^19^ The complex interdependencies between these parameters have driven the development of increasingly sophisticated material design strategies, beginning with the mid-20^th^ century commercialization of Bi_2_Te_3_-based alloys for niche cooling applications and evolving into today’s high-performance systems that push *ZT* boundaries through innovative phonon engineering and band structure manipulation.^20^^,^^21^^,^^22^

The discovery of exceptional thermoelectric performance in tin selenide marked a paradigm shift in the field, demonstrating how synergistic optimization of electronic and thermal transport properties in an Earth-abundant, environmentally benign material could rival the performance of traditional but toxic or rare element-based systems like PbTe or Bi_2_Te_3_.^5^^,^^23^^,^^24^^,^^25^^,^^26^^,^^27^ This breakthrough was enabled by SnSe’s unique combination of anharmonic bonding, layered crystal structure, and temperature-dependent band evolution, which collectively yield ultralow lattice thermal conductivity alongside favorable charge transport characteristics. Although the first improved *ZT* value belongs to single crystal, the translation of these single crystal achievements into practical polycrystalline devices, essential for commercial viability, has exposed three fundamental challenges that continue to limit widespread adoption: (1) the mechanical fragility and anisotropic transport properties of single crystals that complicate device integration and manufacturing scalability; (2) the typically inferior thermoelectric performance of polycrystalline equivalents due to increased *κ*_*latt*_ from grain boundary scattering and degraded *PF* from interfacial carrier recombination; and, most critically, (3) the pronounced asymmetry between well-developed p-type systems and their n-type counterparts due to fundamental material chemistry limitations.^26^^,^^28^^,^^29^ Later, higher *ZT* values were achieved in p-type polycrystalline SnSe as compared to single crystal.^26^^,^^30^ This p-type preference originates from intrinsic Sn vacancies that act as acceptors, creating a self-doping effect that must be overcome through deliberate defect engineering, a challenge that has inspired diverse approaches ranging from stoichiometric control to isovalent/alloying substitutions and interstitial doping, each with distinct impacts on carrier concentration, band structure, and phonon transport.^31^^,^^32^^,^^33^

In addressing these challenges, recent advances in polycrystalline SnSe have bifurcated along two parallel research fronts: p-type optimization and n-type development.^34^ The practical implementation of high efficiency thermoelectric devices requires pairing with complementary p-type materials, making the advancement of high-performance n-type polycrystalline SnSe critically important.^35^^,^^36^^,^^37^ Yet, even after substantial research efforts, the maximum *ZT* value for n-type polycrystalline SnSe still falls short of 2.3 far below the benchmarks set by SnSe single crystals and p-type polycrystalline variants.^38^ This performance gap is primarily attributed to excessive thermal conductivity and a suboptimal power factor in n-type polycrystalline systems.^39^ In stark contrast, n-type SnSe development has proven more complex due to the dual requirements of overcoming intrinsic p-type dominance while simultaneously optimizing electronic and thermal transport properties.^38^ Recent advances in n-type SnSe thermoelectric have relied on strategic approaches to optimize carrier concentration and reduce thermal conductivity. Controlling stoichiometry by introducing Se deficiency or Sn excess helps suppress native Sn vacancies, thereby improving electronic transport.^28^^,^^29^^,^^40^^,^^41^^,^^42^ Another effective route involves extrinsic doping with electron-donating elements, such as halogens substituting at anion sites, which fine-tunes the Fermi level and enhances carrier density.^29^^,^^41^^,^^43^^,^^44^^,^^45^^,^^46^ Various fabrication methods including solid-state reaction,^38^^,^^39^^,^^44^^,^^47^^,^^48^ solvothermal synthesis,^49^ spark plasma sintering (SPS),^48^^,^^50^^,^^51^^,^^52^ and mechanical alloying^53^^,^^54^^,^^55^ have been employed to optimize thermoelectric performance. Halogen substitutions (I, Br, and Cl) have shown particular promise, not only by supplying n-type carriers but also by favorably reshaping the conduction band to boost mobility.^29^^,^^40^^,^^41^^,^^46^ Despite progress, n-type SnSe still trails p-type performance due to unresolved challenges in defect dynamics, dopant stability, and microstructure-property relationships. Bridging this gap demands integrated approaches combining advanced characterization, modeling, and precision synthesis.

This review presents a critical synthesis of contemporary understanding in n-type SnSe thermoelectrics, organized through three principal analytical dimensions: first, we establish the fundamental material challenges through crystallographic and defect chemistry perspectives, elucidating the intrinsic limitations governing carrier transport and thermal conductivity. Second, we conduct a systematic evaluation of fabrication methodologies, bulk processing routes, emerging nanostructured approaches, and their respective impacts on microstructural evolution. Third, we present a comprehensive taxonomy of doping strategies, analyzing mechanistic distinctions between halogen-based, rare-earth, and transition metal dopant systems. The review concludes by delineating persistent knowledge gaps in texture control and interfacial engineering, while proposing a research framework integrating advanced characterization, computational material design, and heterostructure engineering to advance n-type SnSe performance toward practical device implementation.

## Atomic-scale engineering of n-type SnSe

### Structural framework of SnSe

**Figure 1:**
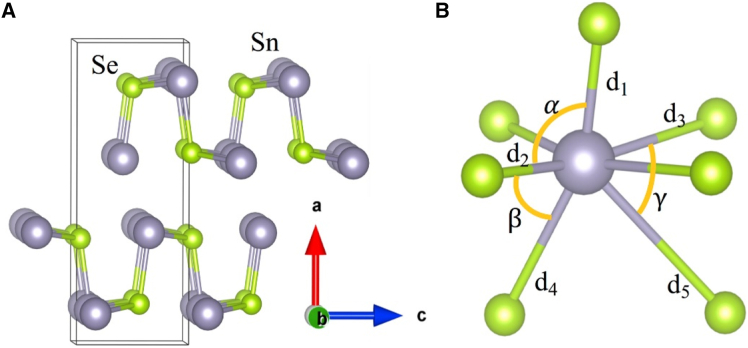
Crystal structure and bonding of SnSe (A) SnSe’s crystal structure and (B) Sn polyhedral coordination (reprinted from Wu et al.^58^, Copyright 2018, with permission from Elsevier).

SnSe adopts an orthorhombic GeS-type structure (Pnma space group at room temperature) characterized by distinctive corrugated layers.^56^ These layers consist of two-atom-thick SnSe slabs stacked along the *a* axis, with selenium and tin atoms occupying separate planes, creating an inherently anisotropic arrangement.^57^ This results in a puckered atomic arrangement featuring zigzag projections along the *b* axis and armchair-like configurations along the *c* axis (Sn-Se chains), as illustrated in Figure 1A.^58^ The interlayer bonding exhibits weak van der Waals-like character, while stronger covalent interactions dominate within the layers.^59^ A notable reversible phase transition occurs between 600 and 800 K, transforming the structure to a higher-symmetry Cmcm arrangement, as confirmed by advanced characterization techniques.^60^ This transition manifests through changes in the Se intralayer-to-interlayer distance ratio, correlating with enhanced symmetry and improved charge carrier mobility.

### Bonding characteristics and electronic structure

Figure 1B illustrates the low-temperature *Pnma* phase of SnSe, highlighting the anisotropic coordination environment of Sn^2+^ centers. The Sn atoms adopt a distorted polyhedral structure, coordinated by Se atoms through distinct Sn-Se bonds; the distances d_1_, d_2_, d_3_, d_4_, and d_5_ are showing the Sn-Se bonding connections. The polyhedral distortion is further quantified by bond angles α, β, and γ, which deviate significantly from ideal geometry.^58^ This distortion originates from the stereochemically active 5s^2^ lone electron pair on Sn^2+^, which contributes to pronounced bonding anharmonicity—a crucial factor influencing thermal transport properties.^58^ The electronic structure shows remarkable doping dependence: p-type SnSe displays charge localization within layers, while n-type variants exhibit delocalized interlayer conduction pathways due to specific p-orbital interactions near the conduction band minimum.^24^ This fundamental understanding of SnSe’s bonding nature provides a basis for comparison with other layered chalcogenides and related materials systems.

### Computational design of n-type SnSe

**Figure 2:**
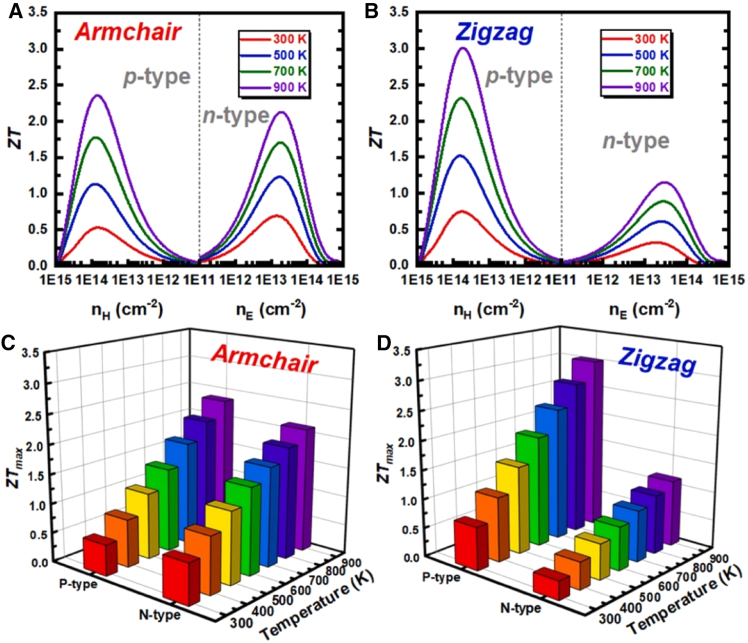
Comparison of P-type and N-type *zT* in armchair and zigzag configuration (A and B) Evolution of *zT* with carrier density from 300 to 900 K, and (C and D) highest achievable *zT* for p-type and n-type SnSe monolayers in armchair and zigzag configurations (reprinted fromWan et al.^61^, Copyright 2024, with permission from Elsevier).

Recently, first-principles calculations using the Vienna *ab initio* Simulation Package with the Perdew-Burke-Ernzerhof functional and projector augmented wave pseudopotentials, incorporating Grimme’s DFT-D3 empirical correction, revealed the exceptional thermoelectric performance of n-type wrinkled SnSe monolayers. Structural relaxations of the 8 × 8 × 1 supercell demonstrated how the 1.56 Å buckling height breaks symmetry, while ShengBTE calculated lattice thermal conductivity (*κ*_*latt*_ = 1.42 W/mK, using second-order interatomic force constants from 2 × 2 × 1 k-grids) and TransOpt derived electronic transport properties (via constant electron-phonon coupling approximation) and quantified the synergy between phonon scattering and multivalley conduction (25–162 meV separations). Crystal orbital Hamilton population analysis confirmed weak Sn-Se antibonding states, and *ab initio* molecular dynamics at 900 K validated stability. Obtained results along armchair and zigzag directions are displayed in Figures 2A and 2B) for *zT* versus carrier concentration across 300–900 K, and Figures 2C and 2D present the corresponding maximum *zT* values as a function of temperature for p-type and n-type SnSe monolayers, collectively explaining the record *zT* = 2.13 for n-type SnSe at 900 K at optimal carrier concentration (*n* = 1.9×10^14^ cm^−2^).^61^

Recent computational studies consistently demonstrate the exceptional thermoelectric potential of n-type SnSe. First-principles KKR calculations reveal n-type SnSe’s superior thermoelectric potential, achieving higher power factors along the interlayer x-direction compared to p-type’s in-plane transport. The phase transition at 807 K induces abrupt band-gap changes while preserving anisotropic “pudding-mold” valence bands, with optimal performance above 600 K at heavy doping. Theoretical predictions align with p-type experimental data below 600 K, while n-type shows enhanced interlayer transport ideal for *zT* optimization.^62^ First-principles calculations predicted the exceptional thermoelectric performance (*zT* ≈ 3.1 at 770 K along the *a* axis), due to superior electrical conductivity and Seebeck coefficient, and demonstrated that how Boltzmann transport theory and atomic-scale design can optimize these properties.^63^ Complementing these findings, recent work demonstrates how electron mean free path filtering in n-type SnSe enhances room temperature *zT* to 0.62 (vs. bulk 0.26) by tailoring grain boundaries. Density functional theory (DFT)/Wannier calculations reveal that h-LO phonons dominate electron scattering, while three-phonon processes control thermal transport, enabling selective phonon suppression with minimal electron impact.^64^ Extending this strategy, grain boundary engineering in SnSe yields remarkable *zT* enhancements, boosting n-type to 1.7 along the *xx* axis at 300 K. First-principles calculations reveal that this stems from selective phonon scattering while preserving electronic transport dominated by LO-mode electron-phonon coupling, with negligible coherence thermal contributions.^65^ First-principles analysis reveals that n-doped SnSe achieves *zT* = 3.1 at 807 K due to three synergistic effects: (1) ionized impurity-dominated scattering, (2) higher electronic group velocities enhancing power factor, and (3) rapid *κ*_*ele*_ reduction above 600 K.^66^ Collectively, these studies establish n-type SnSe as a paradigm-shifting thermoelectric material, where strategic manipulation of electronic transport, phonon scattering, and microstructure engineering synergistically overcome traditional performance limitations.

## Fabrication methods used for preparing n-type SnSe

N-type SnSe realized through two primary approaches are discussed here: (1) thin film and nanosheet fabrication for flexible electronics and integrated devices and (2) bulk synthesis followed by consolidation for structural control. Thin films enable low-temperature processing and substrate versatility, while bulk methods permit precise doping and microstructure tuning. Emerging hybrid techniques like 3D printing bridge these paradigms using nanoparticle inks.

### Thin film fabrication

**Figure 3:**
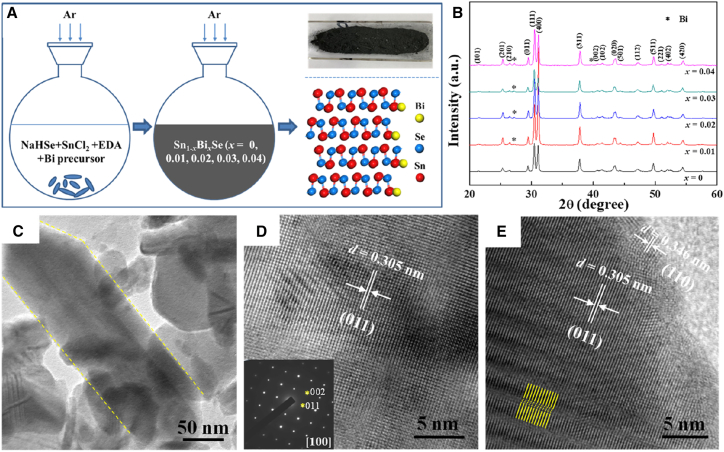
Synthesis and structural characterization of solution-processed Sn_1-x_Bi_x_Se nanomaterials (A) Experimental setup and as-synthesized nanopowders from the wet-chemical reaction. (B) X-ray diffraction patterns confirming the orthorhombic phase (Pnma) for undoped and Bi-doped (x = 0–0.04) compositions. (C) TEM micrograph showing the characteristic plate-like morphology of Sn_0.97_Bi_0.03_Se, with a representative nanoribbon highlighted. (D) [100] HRTEM image with inset SAED pattern, revealing (011) lattice planes (d-spacing = 0.305 nm). (E) Identification of antiphase boundaries (yellow lines) and partial surface oxidation (reprinted with permission from Li et al.^67^ Copyright 2018 American Chemical Society).

The n-type Sn_1-x_Bi_x_Se (x = 0–0.04) was synthesized via a scalable solution method, shown in Figure 3A, where precursors (SnCl_2_/Bi salts/Se) were reduced in ethylenediamine/NaOH/NaBH_4_ at 423 K. X-ray diffraction (XRD) analysis in Figure 3B confirmed the orthorhombic SnSe phase (Pnma, JCPDS 48–1,224) with Bi-induced peak shifts. Transmission electron microscopy (TEM) characterization in Figures 3C–3E revealed: (1) anisotropic nanoplates, (2) single-crystalline [100] zones with (011) lattice fringes (d = 0.305 nm) and SAED-verified crystallinity in Figure 3D, and (3) antiphase domains (Figure 3E, yellow lines) beneficial for phonon scattering. Post-synthesis annealing (H_2_/Ar, 300°C) and sintered densification preserved these nanostructures, achieving >95% relative density for thermoelectric measurements.^67^ N-type Bi-doped SnSe films were fabricated by pulsed laser deposition (248 nm) from pre-sintered targets onto SrTiO_3_(100) substrates at 300°C under high vacuum. Structural characterization employed XRD for epitaxial analysis and STEM-EDX for elemental mapping, while thermoelectric properties were measured via four-probe conductivity, Seebeck coefficient (K-type thermocouples), and Hall effect (PPMS, 3 T).^68^

N-type Sn_0.94_Bi_0.06_Se nanosheets (1.2–3 nm thick) were synthesized via a low-temperature solution route using SnCl_4_·5H_2_O, SeO_2_, and Bi-neodecanoate in oleylamine/Phen (200°C, N_2_ atmosphere), achieving 95% yield with controlled morphology. Structural characterization by PXRD, High-resolution transmission electron microscopy (HRTEM) (d_011_ = 0.29 nm), and SAED confirmed phase-pure orthorhombic crystals, while EDAX/XPS verified homogeneous Bi doping (6 mol %). Capping agent removal (500°C/N_2_) was validated by Fourier transform infrared spectroscopy. Sintering-produced dense pellets were also fabricated for anisotropic thermoelectric measurements, revealing enhanced n-type conductivity (Hall) and reduced lattice thermal conductivity parallel to the pressing direction.^69^

### Bulk synthesis

Bulk n-type SnSe materials are fabricated through a two-stage process: (1) powder synthesis (solvothermal, arc melting, mechanical alloying, etc.) and (2) densification (SPS, hot pressing, etc.). In the following, we detail the methodologies and trade-offs of these approaches for optimizing thermoelectric performance.

#### Powder synthesis

Various methods are available for synthesizing n-type SnSe powders, each with unique processing advantages. The melting approach involves direct heating of Sn and Se precursors in a sealed environment, yielding polycrystalline SnSe that can be milled into fine powders. Mechanical alloying bypasses high-temperature processing by using ball milling to mechanically react Sn and Se powders at room temperature, producing nanocrystalline SnSe. For solution-based routes, solvothermal synthesis facilitates low-temperature crystallization in a solvent medium, enabling control over particle size and morphology. Rapid bulk synthesis can be achieved through arc melting, where stoichiometric Sn and Se are melted under inert gas and subsequently crushed into powder. Alternatively, the temperature gradient method employs controlled thermal profiles in a sealed ampoule to drive directional growth, resulting in phase-pure SnSe with tailored stoichiometry. These methods^35^ offer flexibility in terms of scalability, crystallinity, and defect engineering for optimizing n-type SnSe properties.

##### Melting

**Figure 4:**
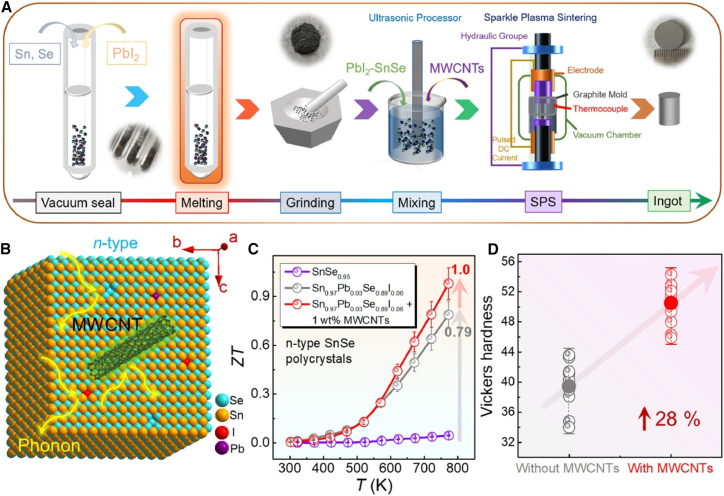
Fabrication and characterization of PbI_2_-doped SnSe/MWCNT composites (A) synthesis schematic; (B) crystal structure illustration; (C) *zT* vs. temperature for SnSe_0.95_, Sn_0.97_Pb_0.03_Se_0.89_I_0.06_, and its MWCNT (1 wt %) variant; (D) comparative Vickers hardness of doped samples with/without MWCNTs (reprinted fromMao et al.^70^, Copyright 2022, with permission from Elsevier).

The n-type polycrystalline SnSe was synthesized via a conventional vacuum melting route as shown in Figure 4A; first three steps are commonly used to get the synthesis powder by the melting, a widely adopted technique for thermoelectric materials due to its simplicity, scalability, and ability to achieve homogeneous phase formation. This method has been particularly dominant in fabricating n-type SnSe, as it ensures precise stoichiometric control and effective dopant incorporation, critical for tuning carrier concentration. In this study, stoichiometric Sn, Se (SnSe_0.95_ to suppress Sn vacancies), and PbI_2_ dopant were sealed in quartz tubes (<10^−3^ Pa) and heated to 1,223 K (100 K h^−1^, 24 h soak). The cooled ingots were ground and uniformly mixed with multi-walled carbon nanotubes (MWCNTs, 0–2 wt %) via ultrasonication, followed by consolidation using SPS at 773 K/55 MPa. As depicted in Figure 4B, the MWCNTs (black threads) bridge adjacent SnSe grains (layered structure), enhancing electron transport while creating phonon scattering interfaces. This synergistic design, combined with Pb/I doping, yielded a peak *zT* of 1 at 773 K as shown in Figure 4C, with hardness (50.5 HV) as shown in Figure 4D, via grain boundary reinforcement.^70^ High-purity Sn (99.9%), Se (99.9%), and CeCl_3_ (99.99%) powders were mixed in nominal compositions of SnSe_0.95_-x mol % CeCl_3_, sealed in quartz tubes under vacuum, and heated to 1,273 K for 10 h in a muffle furnace. The resulting ingots were ground and densified via SPS. Phase composition was verified by XRD (D8 Advance, Cu Kα) with patterns measured parallel and perpendicular to the SPS pressing direction. Field emission scanning electron microscopy (JSM-7500F) analyzed fracture surfaces, while electron probe microanalysis (EPMA, JXA-8230) and energy-dispersive X-ray spectrometry (EDS, JXA-8230) characterized element distribution on polished surfaces.^50^

High-purity Sn (99.999%), Se (99.999%), SnCl_2_ (99.99%), and Pb (99.99%) were reacted in evacuated silica tubes at 1,223 K for 6 h to produce Sn_1.05-x_Pb_x_Se_0.95_Cl_0.05_ ingots. The ingots were ground (<45 μm) and densified via SPS (SPS-211Lx: 783 K, 50 MPa, 5 min) to create melt-synthesized samples. For anisotropy reduction, selected samples underwent ball milling (250 rpm, 24 h) and annealing (473 K, 4 h, Ar) before SPS, producing ball-milled/annealed (BA) samples.^42^ Building on the established melting synthesis approach for n-type SnSe, multiple Sn purification cycles and double-sealed tubes were implemented to prevent oxidation. Sn chunks were purified via three cycles of heating in evacuated silica tubes (623 K, 3 h, 10^−4^ Torr) until no black oxide residues remained. In an Ar glovebox (O_2_ < 1 ppm, H_2_O 0 ppm), stoichiometric mixtures (∼13 g) were loaded into inner silica tubes (10^−4^ Torr), which were double-sealed within larger evacuated tubes to prevent oxidation if the inner tube cracked during cooling. These assemblies were heated to 1223 K (9 h ramp, 12 h soak) in a furnace and water-quenched. Resulting ingots were ground and densified by SPS.^38^ High-purity raw materials were used to prepare n-type SnSe crystals with Te and Mo doping. Stoichiometric mixtures were vacuum-sealed in quartz tubes, heated to 1313 K for 16 h, and maintained at 1313 K for 16 h to obtain ingots. The ingots were ground and sealed in pointed-bottom quartz tubes for crystal growth. Tubes were placed in a vertical furnace with the middle at 1313 K and the bottom ∼50 K cooler, creating a temperature gradient. After 16 h at 1313 K, the furnace cooled to 1073 K at 1 K/h before natural cooling to room temperature.^47^ The described melting protocol follows established methodologies for n-type SnSe preparation, with analogous high-temperature synthesis approaches being widely employed across the literature.^28^^,^^39^^,^^40^^,^^41^^,^^43^^,^^44^^,^^45^^,^^46^^,^^48^^,^^51^^,^^52^^,^^67^^,^^71^^,^^72^^,^^73^^,^^74^^,^^75^^,^^76^^,^^77^^,^^78^^,^^79^^,^^80^^,^^81^^,^^82^^,^^83^

##### Mechanical alloying

**Figure 5:**
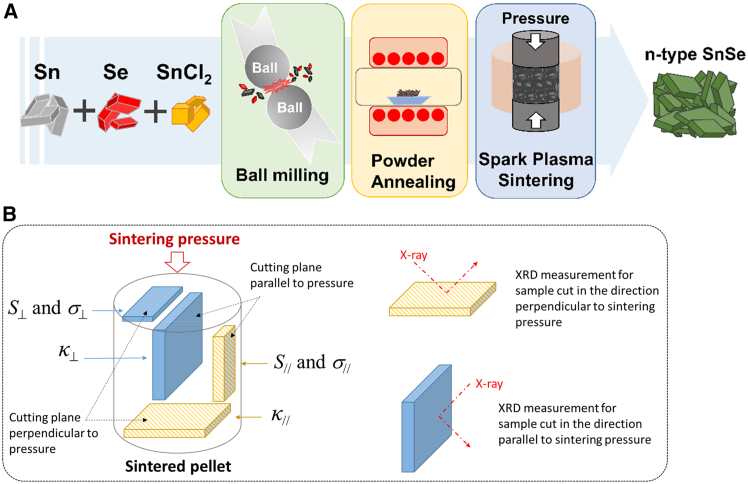
Schematic diagrams showing (A) Process flow for preparing n-type polycrystalline SnSe bulk samples via ball milling, pre-annealing, and SPS consolidation; (B) configuration of samples for thermoelectric and XRD characterization in perpendicular (⊥) and parallel (//) directions with respect to the applied pressure (reprinted fromViet et al.,^55^ Copyright 2023, with permission from Elsevier).

The n-type SnSe polycrystalline bulk was synthesized via a sequential process of ball milling, pre-annealing, and SPS, as illustrated in Figure 5A. High-purity Se, Sn, and SnCl_2_ powders were mixed in Sn-excessive stoichiometry (SnSe_0.92_) to minimize p-type Sn vacancies, with x wt % SnCl_2_ (x = 0, 2, 4) added as dopant. The mixture was ball-milled in an argon-filled glovebox using an SPEX 8000D Mixer/Mill (4 h total, 15 min active/15 min rest cycles to suppress the increase in temperature) in a stainless steel vial with stainless steel balls. The as-milled powder was vacuum-annealed (523 K, 8 h) to convert carriers to n-type, then sintered by SPS. Following Figure 5B, the sintered pellets were cut and polished into rectangular parallelepipeds for anisotropic thermoelectric measurements, with properties evaluated both perpendicular (⊥) and parallel (//) to the SPS pressure direction.^55^ The precursor powders Sn, Se, SnI_2_ were mechanically alloyed using a QM-3SP2 planetary ball mill at 450 rpm for 10 h under argon atmosphere. The process employed a 500 mL stainless steel vessel containing stainless steel balls of two diameters (6 mm and 12 mm), with a precise 1:20 weight ratio between powder and balls. This extended high energy milling ensured complete homogenization and effective incorporation of iodine dopants into the Sn-Se matrix. The resulting powder was subsequently processed by SPS to produce dense n-type SnSe_0.95-x_I_x_ polycrystals for thermoelectric characterization. One of the lowest anisotropic values was achieved by Abbas et al.^29^ High-purity Sn and Se powders were mechanically alloyed in a planetary mill (Retsch PM400) at 300 rpm for up to 20 h (10 min milling/5 min rest cycles) using a 10:1 ball to powder ratio (5 mm SUS304 stainless steel balls, STD11 jar, Ar atmosphere). The alloyed powder was uniaxially compacted (300 MPa) and pressureless-sintered. Structural characterization included XRD for phase analysis and EPMA for compositional mapping. Thermoelectric properties were evaluated via electrical resistivity (Agilent DAQ 34970A) and Seebeck coefficient measurements (Peltier plate apparatus with T-type thermocouples), where the Seebeck coefficient was derived from the slope of open-circuit voltage versus temperature gradient.^53^ In similar way some other n-type SnSe materials were prepared for thermoelectric properties optimization.^42^^,^^54^^,^^67^^,^^84^^,^^85^

##### Solvothermal

The Sb doped SnSe microplates were synthesized via a solvothermal method using Na_2_SeO_3_, SnCl_2_·2H_2_O, and Sb_2_O_3_ precursors dissolved in ethylene glycol (serving as both solvent and reducing agent) with NaOH for pH adjustment. The reaction was conducted in a 125 mL Teflon-lined autoclave at 230°C for 36 h under autogenous pressure, followed by natural cooling to room temperature. The product was collected by centrifugation, washed with ethanol and deionized water, and dried at 60°C for 15 h. Phase purity and crystallinity were confirmed by XRD with lattice parameter analysis using JADE software, while XPS verified the Sb oxidation state. Electron probe microanalysis (EPMA) with 0.1% instrumental error provided precise compositional analysis, confirming the Sb doping level and Se vacancy formation (SnSb_x_Se_1-2x_ stoichiometry). HRTEM and spherical aberration-corrected STEM revealed the single-crystalline nature of the microplates and atomic-scale defects induced by Sb doping. For thermoelectric measurements, the microplates were consolidated into dense pellets via SPS.^49^

##### Arc melting

The synthesis of n-type Sb alloyed SnSe polycrystals was achieved via a well controlled arc melting technique. Stoichiometric mixtures of high-purity Sn, Sb, and Se powders were pelletized under an inert N_2_ atmosphere and subsequently arc melted in a water cooled Cu crucible under Ar to form homogeneous intermetallic ingots. This method ensured precise compositional control (x = 0.1–0.4 in Sn_1-x_Sb_x_Se) while minimizing oxidation. The resulting ingots were either ground into fine powders for structural analysis or cut into bar shaped specimens for transport measurements. The arc melting process proved effective in producing dense, phase-pure polycrystalline samples, which were further characterized to assess their thermoelectric performance, demonstrating its suitability for synthesizing high quality n-type SnSe based materials.^86^ The preparation of high-performance n-type SnSe polycrystals was carried out using a controlled arc melting process in an Edmund Buhler MAM-1 mini-arc furnace under an argon atmosphere. Stoichiometric mixtures of high-purity Sn and Se were ground, pelletized, and arc melted in a water cooled Cu crucible to form dense intermetallic ingots. Unlike SPS, which can introduce detrimental oxide layers at grain boundaries, the arc melting method directly produced mechanically robust pellets with strong intergranular bonding. These ingots were either ground into powder for structural analysis or cold pressed into dense (∼90% crystallographic density) pellets for thermoelectric measurements, ensuring reproducibility and ease of characterization. This synthesis approach effectively minimized extrinsic defects while maintaining high sample integrity, making it a reliable method for producing high quality n-type SnSe thermoelectric materials.^87^

##### Temperature gradient

The n-type SnSe single crystals were synthesized using a controlled vertical temperature gradient method, ensuring high crystallinity and phase purity. Stoichiometric mixtures of high-purity Sn and Se with PbBr_2_ dopant (x = 0–3 mol %) were sealed in double walled quartz tubes under high vacuum (∼10^−5^ Pa) to prevent oxidation and accommodate thermal expansion. The tubes were heated to 1223 K in a rocking furnace for homogenization, followed by quenching to form precursor ingots. For crystal growth, the crushed precursor was resealed in evacuated quartz tubes and placed in a vertical gradient furnace, where it was heated to 1223 K (20 h), soaked (10 h), and slowly cooled to 973 K over 250 h (0.1 K/min) to promote single crystal nucleation. A final cooling step to room temperature (20 h) completed the process. This gradual cooling regime, combined with the axial thermal gradient, minimized defects and enabled the growth of large, high quality single crystals suitable for anisotropic thermoelectric characterization. The method’s reproducibility and effectiveness in controlling dopant distribution were confirmed through XRD and TEM analysis.^88^ Influence of key synthesis methods on the thermoelectric performance, scalability and material quality of n-type polycrystalline SnSe is presented in Table 1.

**Table 1:** Analysis of synthesis methods’ influence on the performance, scalability, and material quality of n-type polycrystalline SnSe

Method	Performance & scalability	Material quality	Key references
**Melting**	excellent for homogeneous doping and high *zT*.^39^ Simple, scalable for large ingot production.	high purity and crystallinity, can lead to large grains. Risk of oxidation and Se volatilization.	Gong et al.^39^; Nisar et al.^40^; Ge et al.^41^; Cha et al.^42^; Li et al.^43^
**Mechanical alloying**	effective for nanocrystalline powders and dopant incorporation, achieves high *zT* with Iodine and reduces anisotropy.^29^ Room temperature technique, scalable via industrial ball mills.	fine, homogeneous powders. Risk of contamination from milling media.	Abbas et al.^29^; Byun et al.^53^; Gu et al.^54^; Viet Chien et al.^55^
**Solvothermal**	excellent control over particle morphology, good for dense SPS pellets. Batch process, limited scalability by autoclave size.	high phase purity and unique morphologies. May involve organic residues.	Shi et al.^49^
**Arc Melting**	produces dense, phase-pure ingots directly. Effective for homogeneous alloying. Fast process scalability for small batches.	very dense, robust pellets with strong intergranular bonding. Minimizes oxide layers.	Gainza et al.^86^; Gainza et al.^87^

#### Densification

Multiple techniques transform n-type SnSe powders into dense bulk materials with tailored properties. SPS rapidly achieves >98% density in minutes while preserving fine grains to reduce thermal conductivity. Hot pressing combines heat and pressure to align grains, enhancing anisotropic electrical transport. Hot forging further improves texture through directional stress, boosting carrier mobility in specific crystal directions. For scalable production, pressureless sintering offers a simpler approach, typically reaching 85–90% density. These methods enable independent optimization of electronic and thermal transport for peak thermoelectric performance.^35^

##### Spark plasma sintering

SPS enables rapid consolidation of powders through simultaneous application of pulsed electric current and uniaxial pressure as schematically shown in Figures 4A and 5A. In recent study mechanically alloyed SnSe powders were consolidated using SPS in a Labox-325 system. The powders were loaded into a graphite die (10 mm inner diameter, 30 mm height) and sintered at 773 K for 15 min under vacuum. During sintering, a uniaxial pressure of 50 MPa was applied to ensure proper densification. This process yielded dense cylindrical pellets (Ф10 × 8 mm) suitable for comprehensive characterization as schematically shown at the right side of Figure 4A. Prepared pallets can be cut in specific way for different characterizations and performance measurements as shown in Figure 5B. Which enabled systematic measurements of electrical and thermal transport properties in both parallel and perpendicular directions, facilitating evaluation of thermoelectric performance.^29^ This technique is very common in fabrication of n-type SnSe thermoelectric materials sometimes minor changes in parameters like sintering temperature, pressure, time as reported in literature.^28^^,^^38^^,^^39^^,^^43^^,^^48^^,^^50^^,^^51^^,^^52^^,^^54^^,^^55^^,^^70^^,^^72^^,^^73^^,^^74^^,^^76^^,^^79^^,^^80^

##### Hot press

Hot pressing shares similarities with SPS, including the use of graphite dies and uniaxial pressure, but differs fundamentally in its external resistive heating mechanism and longer processing times (1 h vs. minutes in SPS). For ZnCl_2_ doped SnSe, pre-synthesized ingots were ground and loaded into a 10 mm graphite die, then consolidated at 760 K under 50 MPa in an argon atmosphere for 1 h. Unlike SPS where rapid Joule heating and plasma activation enable fast densification, hot pressing relies on gradual heat transfer from the furnace environment, requiring extended dwell times to achieve full density while minimizing thermal stress. This approach preserved the SnSe phase stability and yielded dense 10 mm diameter pellets suitable for thermoelectric characterization, with controlled grain growth and reduced risk of compositional decomposition compared to faster sintering methods. For comprehensive characterization, these bulk samples were cut into two geometries: (1) cuboid specimens (2.2 × 2.2 × 8.0 mm^3^) for simultaneous electrical conductivity and Seebeck coefficient measurements using an LSR-3/1100 instrument, and (2) thin discs (10.0 × 1.5 mm) for thermal diffusivity analysis via LFA457 instrument.^44^ Similar hot pressing approaches have been successfully employed in other studies of n-type SnSe thermoelectric materials, demonstrating the reliability of this consolidation method.^75^^,^^77^^,^^81^^,^^83^^,^^84^^,^^89^

##### Hot forge

Hot forging was employed as a critical secondary processing step to induce strong crystallographic texture in the Pr-doped SnSe polycrystals. The sintered disks were loaded into a larger graphite die and subjected to uniaxial pressing at 873 K for 5 min under 40 MPa pressure, significantly enhancing grain alignment along the pressing direction. This deformation process improved charge carrier mobility while reducing lattice thermal conductivity through phonon scattering at textured grain boundaries, as confirmed by XRD orientation factor analysis using the Lotgering method. The combined effects of hot pressing followed by hot forging ultimately optimized the thermoelectric performance by simultaneously enhancing electrical transport and suppressing thermal conduction in the preferred crystallographic direction.^84^

##### Pressureless sintering

Another study employed pressureless sintering as a key consolidation method for SnSe polycrystals, offering distinct processing advantages. The mechanically alloyed powders, compacted at 300 MPa, were sintered at 550°C for up to 120 min under flowing argon (0.7 L/min), producing cylindrical samples (13 mm diameter × 1 mm thickness) without external pressure. This technique enabled controlled phase formation and stoichiometric preservation while avoiding the complexity of pressure-assisted methods. Notably, the argon atmosphere prevented selenium volatilization, and the extended sintering duration facilitated complete phase transformation from mechanically alloyed precursors to crystalline SnSe, as confirmed by XRD analysis. The pressureless approach also promoted surface depletion effects, which contributed to the observed p-type to n-type conversion, a critical factor in enhancing thermoelectric performance through optimized carrier concentration. Compared to hot pressing or SPS, this method provided a simpler, scalable route for fabricating SnSe with tailored electronic properties, while maintaining sufficient density for thermoelectric measurements (validated through EPMA and Raman spectroscopy).^53^ Influence of key sintering methods on the thermoelectric performance, scalability and material quality of n-type polycrystalline SnSe is presented in Table 2.

**Table 2:** Analysis of sintering methods’ influence on the performance, scalability, and material quality of n-type polycrystalline SnSe

Method	Performance & scalability	Material quality	Key references
**SPS**	most common method, preserves nanoscale features, enables strong texturing. Fast cycle times, limited by die size.	high density, textured microstructures. Possible carbon contamination.	Liu et al.^50^; Chandra et al.^51^; Yadav et al.^52^; Zhang et al.^72^; Shi et al.^73^; Taneja et al.^74^
**Hot pressing**	similar to SPS but with external heating, achieves high density and good performance. Slower than SPS.	good density and texture. Lower risk of contamination.	Wu et al.^44^; Shen et al.^75^; Cai et al.^77^; Li et al.^83^; Li et al.^89^
**Hot forge**	secondary process to enhance texture, boosting carrier mobility and *zT*. Additional processing step.	maximizes crystallographic alignment.	Li et al.^84^
**Pressureless sintering**	induces p-to-n transition via Se depletion.^53^ Simple furnace process.	lower density, more porosity. Higher risk of secondary phases.	Byun et al.^53^

### Advanced methods

**Figure 6:**
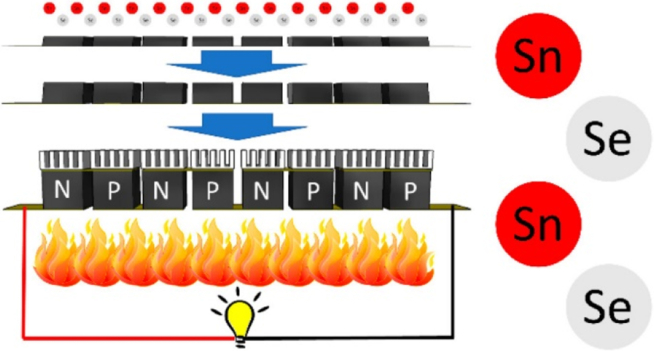
Schematic of eight-leg thermoelectric generator Reprinted fromBurton et al.^90^ under the terms of a Creative Commons CC BY License Published 2023 American Chemical Society

This study demonstrates the viability of 3D printing as a novel fabrication method for SnSe thermoelectric materials, offering advantages in complex geometry control and scalable production. The pseudo-3D printing technique employed a customized ink formulation containing ball-milled SnSe-Bi powder (37 g) blended with a sodium carboxymethylcellulose binder solution (13 g). Layer-by-layer deposition into a 3D printed ABS sacrificial mold on a 120°C hotplate created structures with approximately 2 mm thick layers, reaching a final leg height of 1 cm. The printed elements underwent controlled curing (heating to 873 K at 0.5 K/min followed by natural cooling) to achieve dense, functional thermoelectric materials. Subsequent XRD and SEM characterization confirmed the preserved thermoelectric properties, while the method’s versatility was demonstrated through fabrication of a complete eight-leg thermoelectric generator incorporating (Figure 6) both n-type (8% Bi doped) and p-type SnSe elements with optimized copper connections and aluminum heat sinks.^90^

## Doping strategies for enhanced thermoelectric performance

The thermoelectric performance of n-type SnSe manifests in both its intrinsic form and through doping modifications. This section examines: the intrinsic properties of undoped SnSe, halogen doping effects (Cl, Br, I), other doping effects including rare-earth and transition metal doping etc. The discussion focuses on how each system contributes to thermoelectric performance through its distinct mechanisms.

### Undoped SnSe

The undoped n-type SnSe polycrystals exhibit remarkable intrinsic thermoelectric performance, transitioning from p-type to n-type behavior above 580 K with a peak Seebeck coefficient of −350 μV/K. Neutron diffraction confirms this n-type character stems from a 3% Sn deficiency (Sn_0.969_Se), while XPS reveals surface SnO_2_ formation that modifies grain boundary conductivity. The material achieves ultralow thermal conductivity (0.37 Wm^−1^K^−1^ at 873 K) and a record *zT* ∼1.8 at 816 K for undoped polycrystalline SnSe, driven by two key mechanisms: (1) the orthorhombic to Cmcm phase transition at 795 K that enhances electrical transport, and (2) phonon softening observed via inelastic neutron scattering that suppresses heat conduction. These intrinsic properties demonstrate that undoped SnSe can rival doped variants in high-temperature applications.^87^ The n-type polycrystalline SnSe exhibited dynamic thermoelectric properties strongly influenced by sintering duration, showing a remarkable p-type to n-type transition with Seebeck coefficient reversal from +503 μV/K to −1500 μV/K. This conduction type conversion, attributed to Se depletion creating electron dominant carriers, was accompanied by significant electrical conductivity variations, initially increasing with densification (10-min sintering) then decreasing due to SnO_2_ formation (120 min sintering). Compositional analysis revealed progressive Se depletion from surface to bulk, eventually achieving uniform Sn/Se ratios after 120 min while developing SnSe_2_ and SnO_2_ secondary phases. Notably, prolonged sintering caused reversion to p-type behavior as interfacial SnO_2_/SnSe potential barriers induced energy filtering. These findings demonstrate how sintering controlled stoichiometry modifications and phase evolution can strategically manipulate charge transport in polycrystalline SnSe systems.^53^

**Figure 7:**
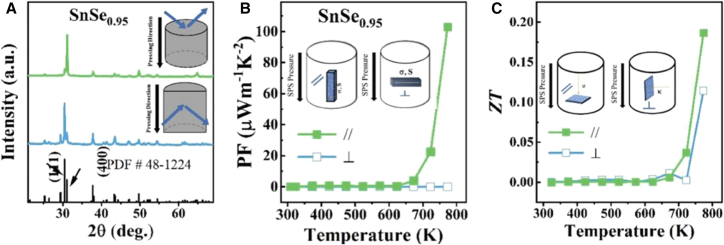
Structural and thermoelectric anisotropy in SnSe_0.95_ polycrystals (A) XRD patterns parallel (//) and perpendicular (⊥) to the SPS pressure direction compared with standard reference (PDF # 48 - 1224), showing characteristic (111) and (400) peak variations that confirm Pnma phase purity and crystallographic anisotropy, (B) temperature-dependent power factor demonstrating enhanced performance in the pressure aligned (//) direction, and (C) dimensionless figure of merit (*zT*) values revealing superior thermoelectric efficiency along the//orientation, particularly at elevated temperatures (reprinted fromNisar et al.,^40^ Copyright 2024, with permission from John Wiley and Sons).

The n-type SnSe polycrystals were engineered through precise stoichiometric control (SnSe_0.95_) to minimize Sn vacancies, with phase purity confirmed by XRD patterns collected parallel (//) and perpendicular (⊥) to the SPS pressure direction as shown in Figure 7A. These patterns matched the standard Pnma structure (PDF # 48–1224), while the distinct (111) and (400) peak intensities revealed pronounced crystallographic anisotropy. Thermoelectric characterization demonstrated strong directional dependence, with the//direction exhibiting superior performance, achieving both higher power factor and enhanced *zT* values as shown in Figures 7B and 7C, respectively compared to the ⊥ orientation, particularly at elevated temperatures. This marked anisotropy guided the selection of the//direction for subsequent optimization studies.^40^

### Halogen co-doping

Halogen co-doping (Cl, Br, I) has emerged as a pivotal strategy for optimizing the thermoelectric performance of n-type SnSe by tailoring its electronic structure and charge transport properties. This section systematically examines the distinct roles of chlorine (Cl), bromine (Br), and iodine (I) as co-dopants, highlighting their impacts on electrical conductivity, Seebeck coefficient, and thermal conductivity. While all three halogens promote n-type conduction, their efficacy varies due to differences in ionic radii, electronegativity, and bond energetics, which collectively influence defect formation and band convergence. This section examines how these dopants modify the material’s electronic and thermal transport properties based on experimental and theoretical studies.

#### Chlorides co-doping

Chlorine doping in n-type SnSe is primarily achieved through chloride based precursors rather than elemental chlorine. These dopants not only tune charge carrier concentration but also modify defect chemistry, influence phonon scattering, and alter band structure, all of which can significantly impact thermoelectric performance. Representative cases demonstrating these multifaceted effects are discussed here.

**Figure 8:**
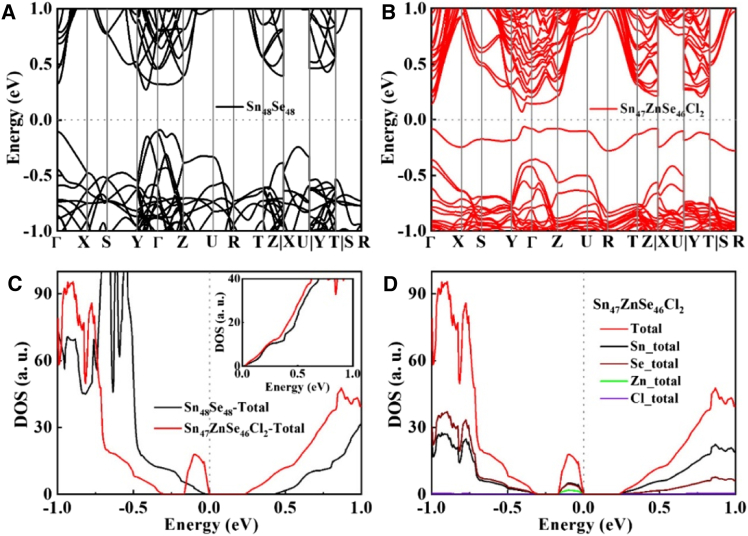
Electronic structure of pristine and doped SnSe Electronic band structure and density of states (DOS) for (A) pristine Sn_48_Se_48_ and (B) ZnCl_2_ doped Sn_47_ZnSe_46_Cl_2_, with (C) total DOS comparison and (D) element-projected DOS of the doped system (reprinted with permission from Wu et al.^44^ Copyright 2018 American Chemical Society)

Cl-based dopants (e.g., HfCl_4_, NbCl_5_, MoCl_5_, NdCl_3_/CeCl_3_, Zr/Cl) and SnCl_2_ processing significantly enhance the thermoelectric performance of n-type SnSe, achieving *zT* values up to ∼1.42, arises from optimized carrier concentration, multipoint defect engineering, and scalable n-type conversion.^55^^,^^72^^,^^73^^,^^75^^,^^77^^,^^79^ ZnCl_2_ doping in n-type polycrystalline SnSe demonstrates remarkable thermoelectric enhancement through synergistic band and defect engineering. The optimized SnSe_0.95_–2% ZnCl_2_ achieves a peak *zT* ∼ 1.3 at 873 K, a four-order-of-magnitude improvement over pristine SnSe, attributed to (1) boosted electrical conductivity via elevated carrier concentration (∼1.3 × 10^19^ cm^−3^), (2) a high Seebeck coefficient (∼421 μVK^−1^ at 873 K) enabled by multivalley band convergence, and (3) ultralow lattice thermal conductivity from phonon scattering by ZnSe precipitates, amorphous Sn-Cl phases, and twin boundaries. DFT calculations reveal the electronic origins of these improvements: Comparative analysis of Sn_48_Se_48_ (pristine) and Sn_47_ZnSe_46_Cl_2_ (doped) supercells as shown in Figures 8A and 8B, respectively present that ZnCl_2_ doping introduces impurity levels near the Fermi level, creating a localized peak in the density of states (DOS) as in Figure 8C. This peak arises primarily from orbital hybridization between Sn/Se p-electrons and Zn s - electrons as in Figure 8D, enhancing the density of states effective mass and Seebeck coefficient. Additionally, ZnCl_2_ doping slightly increases DOS near the conduction band minimum via multivalley convergence, further optimizing electrical transport. These electronic structure modifications, combined with defect-mediated phonon scattering, underscore Cl’s dual role as both an electronic dopant and microstructure modifier in n-type SnSe.^44^

CeCl_3_ doping in n-type SnSe enhances thermoelectric performance through dual mechanisms: (1) increased electron concentration via impurity level excitation, and (2) reduced lattice thermal conductivity (0.29 Wm^−1^K^−1^) through point defect phonon scattering. The anisotropic textured structure optimizes power factor (∼5.63 μWcm^−1^K^−2^) along the parallel direction, achieving peak *zT* ∼ 1.17 at 773 K.^50^ Co doping SnSe with Cl and PbSe achieves record n-type performance with *zT* ∼ 1.2 at 823 K, combining enhanced power factor (∼6.8 μW cm^−1^ K^−2^) and reduced lattice thermal conductivity (∼0.64 W m^−1^K^−1^). Post processing eliminates anisotropy, yielding isotropic *zT* for scalable applications.^42^ SnCl_2_ and Cu co doping in n-type SnSe achieves a peak *zT* of 1.55 at 773 K through optimized carrier concentration (4.2×10^18^ cm^−3^) and ultralow lattice thermal conductivity (0.29 Wm^−1^K^−1^). The composite simultaneously enhances mechanical hardness (1.5 GPa) via Cu_6_Sn_5_ dispersion strengthening while maintaining excellent thermal stability.^43^ NdCl_3_ doping in n-type SnSe boosts electrical conductivity to 37.8 S cm^−1^ (773 K) and power factor to 651 μWm^−1^K^−2^, achieving *zT* ∼ 0.9. Co doping with heavy Bi further reduces lattice thermal conductivity to 0.37 Wm^−1^K^−1^, yielding peak *zT* ∼1.07 (773 K) via multi-scale phonon scattering from secondary phases.^48^ Re and Cl co doping in n-type SnSe achieves a record *zT* ∼ 1.5 at 798 K through synergistic defect engineering. ClSe acts as an n-type donor (*σ* = 50 Scm^−1^), while ReSn point defects and nanoprecipitates reduce lattice thermal conductivity to 0.38 Wm^−1^K^−1^. The optimized carrier concentration yields a high power factor (∼600 μWm^−1^K^−2^) via balanced Seebeck (−450 μVK^−1^) and electrical conductivity.^41^ MoCl_5_ doping in n-type SnSe achieves a record *zT* ∼ 2.0 at 798 K by simultaneously boosting carrier concentration (5.2 × 10^19^ cm^−3^) via Cl^−^ substitution and reducing lattice thermal conductivity to 0.26 Wm^−1^K^−1^ through Mo/Cl induced modular nanostructures (1.2–2.6 nm periodicity).^51^

**Figure 9:**
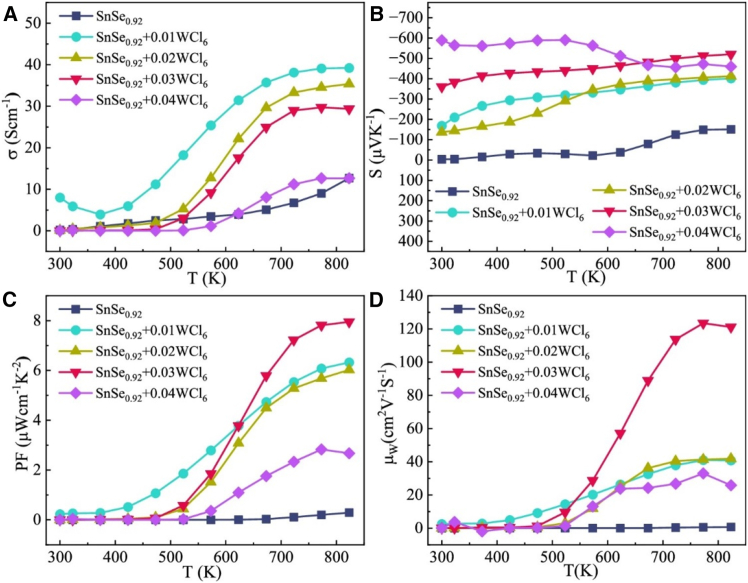
Charge transport characteristics of WCl_6_ doped SnSe (A) *σ*, (B) *S*, (C) PF, (D) *μ*_*W*_ (weighted mobility) along pressing direction. Reprinted fromGong et al.^39^ under the terms of a Creative Commons CC BY License. Published 2024 Springer Nature.

WCl_6_ doping in n-type SnSe enables breakthrough thermoelectric performance, with the SnSe_0.92_ + 0.03WCl_6_ sample achieving a record *zT* ∼ 2.2 at 773 K. This stems from synergistic effects: (1) As shown in Figure 9A, electrical conductivity peaks at 39.2 Scm^−1^ (823 K) for the 0.01WCl_6_ sample (tripling pristine values), while the optimal 0.03WCl_6_ composition maintains high electrical conductivity with dramatically enhanced Seebeck coefficients as presented in Figure 9B, yielding a power factor of 7.95 μWcm^−1^K^−2^ as in Figure 9C, (2) Weighted mobility analysis in Figure 9D confirms band structure modification via W-induced resonance levels and (3) Divacancies (Se/Sn vacancies) and W/Cl rich nanoprecipitates reduce lattice thermal conductivity to 0.24 Wm^−1^K^−1^. Cl plays dual roles here as an n-type dopant (Cl^−^ substitution) and defect engineering agent.^39^

#### Bromides co-doping

Bromide doping has emerged as a promising strategy to further optimize the thermoelectric performance of n - type SnSe. Br incorporation whether in its elemental form or as compounds effectively tunes carrier concentration and introduces defect engineering opportunities. These dopants not only enhance electrical conductivity but also help suppress thermal conductivity through targeted phonon scattering.

**Figure 10:**
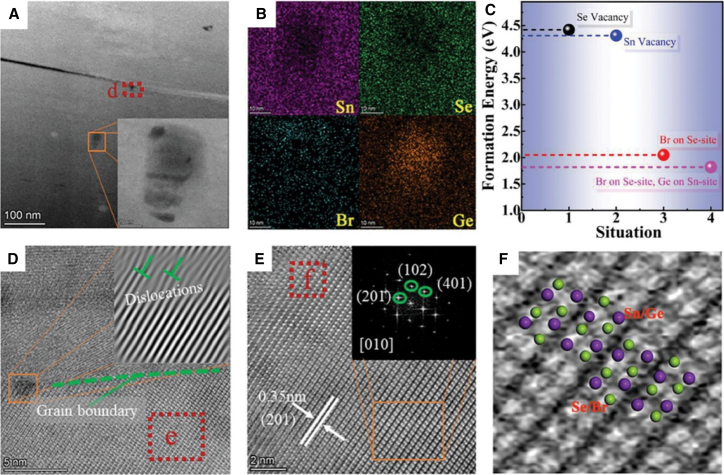
Microstructural and elemental characterization of the Sn_0.99_Ge_0.01_Se_0.91_Br_0.04_ sample (A) TEM image reveals dislocations at grain boundaries (inset: HAADF image highlighting elemental segregation). (B) EDS maps confirm uniform Br distribution and localized Ge rich regions (Sn, Se, Br, and Ge). (C) DFT-calculated formation energies for vacancies and dopant substitutions. (D) HRTEM image of defects (inset: filtered IFFT showing dislocation density). (E) HRTEM of the SnSe matrix (inset: SAED pattern along [010] confirming orthorhombic structure). (F) Atomic-resolution image of Sn/Se lattice arrangement (reprinted fromNisar et al.,^40^ Copyright 2024, with permission from John Wiley and Sons.)

The thermoelectric performance of Sn enriched n-type SnSe polycrystals was significantly enhanced through Br doping and Br/Ge co doping strategies. The pristine SnSe_0.95_ exhibited a modest *zT* of 0.19 and a power factor of 103 μW mK^−2^ at 773 K, with anisotropic behavior favoring the parallel direction relative to the SPS pressure axis. Br doping (SnSe_0.95-x_Br_x_) dramatically improved electrical transport, increasing the *zT* to 0.94 (a 395% enhancement) at 773 K, primarily due to a higher carrier concentration and optimized power factor. However, Br doping alone had minimal impact on thermal conductivity (0.42 Wm^−1^K^−1^). Introducing Ge doping alongside Br (Sn_0.99_Ge_0.01_Se_0.91_Br_0.04_) further optimized the thermoelectric properties: the power factor surged to 662 μW mK^−2^ (543% increase) while lattice thermal conductivity dropped to 0.38 Wm^−1^K^−1^ (11% reduction), yielding a peak *zT* of 1.34, a remarkable 605% improvement over pristine SnSe_0.95_. The microstructural evolution and elemental distribution in Sn_0.99_Ge_0.01_Se_0.91_Br_0.04_ were systematically characterized through TEM analysis (Figure 10). Figure 10A reveals dislocations at grain boundaries, with the HAADF inset showing dopant segregation patterns that initiate phonon scattering. Elemental mapping in Figure 10B demonstrates uniform Br distribution alongside localized Ge rich regions, confirming successful incorporation of both dopants while revealing nanoscale compositional fluctuations. The formation energy calculations in Figure 10C show Br/Ge co doping (1.86 eV) is energetically favorable compared to single doping (2.05 eV for Br), explaining the enhanced dopant incorporation efficiency. High-resolution imaging in Figure 10D exposes a high density of dislocations (verified by IFFT analysis in inset) that effectively scatter phonons. Figure 10E confirms the preserved orthorhombic SnSe matrix (spacing = 0.35 nm) through both lattice imaging and SAED patterns ([010] zone axis), while Figure 10F provides atomic-scale evidence of Sn/Se arrangement and dopant sites, completing the microstructural picture.^40^

(Pb, Br) co doped n-type SnSe polycrystals achieved *zT* = 1.1 at 773K (vs. 0.03 undoped) via 70× carrier boost (1.79 × 10^19^ cm^−3^) and multi nanoprecipitate induced phonon scattering, which reduced lattice thermal conductivity to 0.32 Wm^−1^K^−1^), yielding 532 μWm^−1^K^−2^ power factor.^54^ Br doped n-type SnSe polycrystals achieved *zT* ∼ 1.5 at 783K through optimized carrier concentration (Br: 6%) and textured microstructures. Electrical conductivity surged 6 × (29.5 S cm^−1^) via Br doping, while texturing and excess Sn (Sn_1.005_) reduced lattice thermal conductivity to 0.32 Wm^−1^K^−1^ through cross layer phonon scattering, yielding a 681 μWm^−1^K^−2^ power factor.^85^ Heavy Br doping (10–12%) converted p-type SnSe to n-type, boosting carrier concentration (1.3×10^19^ cm^−3^) and electrical conductivity (800 Sm^-1^). Combined with phonon scattering from Br-induced defects (reduced later thermal conductivity to 0.3 Wm^−1^K^−1^), this yielded a peak *zT* ∼ 1.3 at 773K and higher power factor (4.5 μWcm^−1^K^−2^).^67^ PbBr_2_ doped n-type SnSe crystals achieved a peak *zT* of 2.1 at 770 K along the out of plane direction by strategically modifying Fermi surface dynamics. While optimal doping achieves record-breaking *zT* values, excessive doping distorts the lattice and degrades carrier mobility. Germanium co doping emerges as a corrective solution, restoring the Fermi surface geometry and significantly recovering performance. The work highlights the critical interplay between lattice parameters, Fermi surface evolution, and anisotropic charge transport in achieving high thermoelectric efficiency.^88^ Another study achieved a record high *zT* of ∼2.8 at 773 K in n-type SnSe single crystals along the out of plane direction, marking a significant breakthrough in thermoelectric performance. The exceptional properties stem from three key factors: (1) enhanced electrical transport with 2-fold higher carrier mobility than p-type SnSe, enabled by overlapping interlayer charge density and band divergence during the Pnma to Cmcm phase transition above 600 K; (2) ultralow lattice thermal conductivity of 0.18 Wm^−1^K^−1^ due to strong anisotropic phonon scattering; and (3) optimized band convergence through the temperature induced phase transition, which boosted the *zT* from 2.1 to 2.8. These results demonstrate how crystal structure engineering can simultaneously improve electronic transport while minimizing thermal conductivity in anisotropic materials.^24^

#### Iodides co-doping

While halide doping (Cl, Br) has been widely explored in n-type SnSe, iodide (I) doping remains significantly less studied, with only limited reports investigating its effects on thermoelectric performance. This section examines the unique opportunities and challenges of I co-doping in optimizing carrier transport and phonon scattering in SnSe systems.

**Figure 11:**
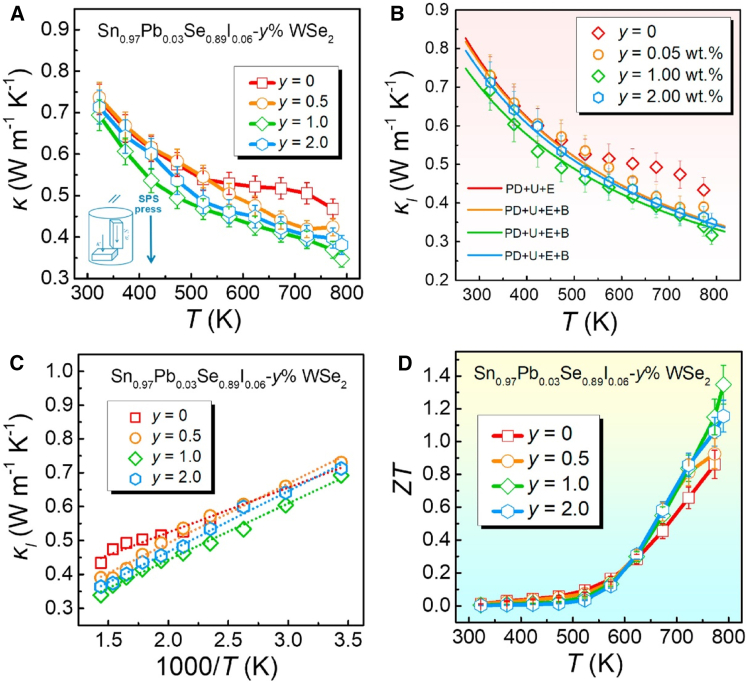
Thermal transport and thermoelectric performance of WSe_2_-enhanced SnSe (A) measured total thermal conductivity, (B) lattice component with theoretical phonon scattering breakdown, (C) temperature variation of lattice thermal conductivity, and (D) resulting thermoelectric figure of merit (*zT*) values. (Reprinted fromChen et al.,^28^ Copyright 2021, with permission from Elsevier).

A peak *zT* of 1.35 at 790 K in n-type polycrystalline SnSe was achieved by incorporating p-type tungsten diselenide (WSe_2_) nanoinclusions into a lead/iodine co doped SnSe matrix. The WSe_2_ inclusions (optimized at 1 wt %) created high density p-n junctions that simultaneously improved electronic properties and suppressed thermal conductivity. These junctions enhanced the Seebeck coefficient (∼470 μV/K) via carrier blocking effects while the 2D WSe_2_ nanostructures reduced lattice thermal conductivity to 0.35 W/m·K through multi-scale phonon scattering. Figure 11 details this thermal transport optimization: (a) Total thermal conductivity shows a 30% reduction with WSe_2_ addition compared to undoped samples. (b) Lattice thermal conductivity analysis using the Callaway model reveals that WSe_2_`s phase boundaries preferentially scatter long wavelength phonons, while point defects (Pb/I doping) target short wavelength phonons. (c) The linear relationship between inverse temperature and lattice conductivity confirms Umklapp scattering remains dominant. (d) The optimal 1% WSe_2_ sample demonstrates a 50% *zT* enhancement over the baseline, attributed to synergistic carrier filtering and phonon scattering. The material maintains structural stability with homogeneous dopant distribution and preserved orthorhombic SnSe phase, as confirmed by XRD and EDS.^28^

Iodine doping successfully converted polycrystalline SnSe to n-type (*zT* ∼ 0.8 at 773K), while 10% SnS alloying further enhanced performance to *zT* ∼ 1.0 by reducing thermal conductivity and optimizing carrier transport. The materials maintained intrinsic ultralow thermal conductivity (<0.5 W/m·K) through anisotropic design and alloy scattering.^45^ Iodine doping successfully converted SnSe to n-type while MWCNT integration enhanced its thermoelectric and mechanical properties. The iodine doping optimized carrier concentration, enabling a *zT* ∼ 1.0 at 773 K, while MWCNTs further boosted electrical conductivity by 40% (45.7 S/cm) and hardness by 28% (50.5 HV), maintaining ultralow lattice thermal conductivity (0.34 W/mK) through interfacial phonon scattering.^70^ A promising strategy to circumvent the characteristic trade-off between the Seebeck coefficient and carrier mobility in thermoelectrics is the deliberate modification of the scattering factor (r). For instance, in n-type SnSe, the engineering of an anisotropic scattering mechanism was shown to enhance both properties concurrently along the out of plane direction. Validated by a quasi-acoustic phonon scattering model, this approach significantly boosted the average figure of merit from 0.84 to 1.57 between 300 and 773 K in I doped samples, underscoring the potential of scattering factor engineering as a vital tool for performance optimization.^91^ Recent study developed weakly anisotropic n-type SnSe through optimized mechanical alloying and iodine doping, achieving a peak *zT* ∼1.02 at 723 K (225% improvement vs. undoped) via synergistic carrier concentration tuning (I doping) and thermal conductivity reduction (0.30 W/mK), while maintaining near isotropic properties (conductivity ratios ∼1) crucial for device integration.^29^

### Other dopings

This section examines alternative doping strategies for SnSe, focusing on chalcogen substitution/complex alloys and rare-earth/transition metal dopants etc., which collectively address electronic band engineering, phonon scattering, and anisotropy control.

#### Chalcogenide substitution/complex alloys

This subsection explores chalcogenide substitution and complex alloying in n-type SnSe, focusing on TaSe_2_ incorporation and (Sn_0.6_Pb_0.4_Se_0.97_Br_0.03_)_0.6_(AgBiSe_2_)_0.4_ composites to simultaneously optimize carrier transport and phonon scattering.

Successful integration of [(SnSe)_1.15_]_7_(TaSe_2_)_1_ ferecrystalline nanostructures within a bulk SnSe matrix was demonstrated for achieving a record thermoelectric figure of merit (*zT* ∼ 2.2 at 823 K) in n-type Ta and Br co doped SnSe_0.92_. The ferecrystals, with their rotational disorder and incommensurate interfaces, drastically reduce lattice thermal conductivity (∼0.18 W/mK at 773 K) by scattering phonons, while Taand Br doping enhances electrical conductivity via increased n-type carrier concentration. HRTEM and HAADF-STEM confirm nm-scale ferecrystalline intergrowths aligned along the *c* direction, combining phonon suppression with efficient charge transport. This approach offers a novel strategy to optimize thermoelectric performance in 2D layered materials by embedding misfit nanostructures in bulk matrices.^74^

Very recent study addresses the poor low-temperature thermoelectric performance of n-type polycrystalline SnSe by stabilizing a cubic phase structure through AgBiSe_2_ alloying, followed by Br doping and Pb alloying. The resulting n-type (Sn_0.6_Pb_0.4_Se_0.97_Br_0.03_)_0.6_(AgBiSe_2_)_0.4_ exhibited significantly improved electrical transport at 300–600 K, achieving a peak *zT* of ∼0.3 at 573 K, surpassing most existing n-type SnSe polycrystals. While the *zT* remains modest, the enhanced low-temperature performance demonstrates the potential for all SnSe based thermoelectric devices. Structural modulation via alloying proved critical in reversing electrical transport behavior, with advanced microscopy confirming atomic arrangements in the cubic lattice.^76^

#### Rare-earth and transition metal doping

Recent studies have demonstrated the effectiveness of rare-earth elements and transition metals in optimizing the thermoelectric performance of n-type SnSe. These dopants, along with their combinations have shown remarkable potential in enhancing carrier concentration while maintaining favorable electronic transport properties. The strategic incorporation of these elements offers a promising approach to improve the power factor and reduce thermal conductivity in n-type SnSe systems.

##### Rare-earth metals

Recent investigations on rare-earth doped SnSe polycrystals have demonstrated significant improvements in thermoelectric performance. For instance, Pr-doped Sn_1-x_Pr_x_Se systems exhibit enhanced n-type behavior, with Sn_0.97_Pr_0.03_Se achieving a peak *zT* of ∼0.7 at 773 K due to increased carrier concentration from Pr^3+^ incorporation at Sn^2+^ sites. Further optimization through hot forging has been shown to improve texturing, reducing thermal conductivity and elevating *zT* to ∼0.9 along the pressing direction. Such advances highlight the critical role of rare-earth doping (e.g., Pr) in simultaneously tuning electronic transport and phonon scattering in n-type SnSe, offering a viable route to overcome the historical limitations of polycrystalline SnSe for mid-temperature thermoelectric applications.^84^

Dual incorporation of rare-earth Ce doping and PbTe alloying significantly enhances the thermoelectric performance of n-type SnSe polycrystals. Ce substitution at Sn sites effectively converts SnSe to an n-type conductor, with Sn_0.97_Ce_0.03_Se achieving a peak *zT* of ∼0.9 at 823 K through improved power factor. PbTe alloying further optimizes the electronic transport by reducing the band gap and increasing both carrier concentration and density of states effective mass, while simultaneously suppressing lattice thermal conductivity via mass and strain fluctuation induced phonon scattering. The synergistic effects yield a record peak *zT* of ∼1.3 at 823 K and an average *zT* of ∼0.52 (300–823 K) in Sn_0.9_Pb_0.07_Ce_0.03_Se_0.93_Te_0.07_. This strategy demonstrates the effectiveness of rare-earth doping combined with chalcogenide alloying in overcoming the intrinsic limitations of polycrystalline SnSe.^78^

##### Transition metals

Transition metal doping with Mo, combined with Te alloying, significantly enhances the in-plane thermoelectric performance of n-type SnSe crystals through crystal symmetry modification. The introduction of Mo and Te increases structural symmetry, boosting carrier mobility to ∼422 cm^2^ V^−1^s^−1^ at 300 K while promoting conduction band convergence for improved electrical transport. This yields a remarkable power factor of ∼28 μWcm^−1^K^−2^. Simultaneously, the Mo and Te create point defects that soften both acoustic and optical phonon branches, reducing lattice thermal conductivity to ∼1.1 Wm^−1^K^−1^ at 300 K. The synergistic optimization of electronic and thermal transport results in exceptional *zT* values (∼0.6 at 300 K and average *zT* ∼ 0.89 from 300 to 723 K) and a record single leg device efficiency of ∼5.3% at ΔT = 300 K.^47^

##### Post-transition metals

Bismuth has emerged as the most frequently employed transition metal dopant for achieving n-type conductivity in SnSe, owing to its ability to overcome thermodynamic doping limitations. Bi-doped n-type SnSe films exhibited characteristic microstructures including ∼200 nm domain boundaries, bimodal Bi precipitates, and stacking faults, which collectively influenced thermoelectric properties. While these structural features initially reduced room temperature mobility, their scattering effects diminished at elevated temperatures, yielding promising n-type behavior with Seebeck coefficients of −385 to −608 μV/K and electrical conductivity of 1.5 S/cm. The self-organized Bi nanoprecipitates, while currently compromising room temperature performance, present significant potential for thermal conductivity reduction, suggesting that optimized control of Bi doping levels and microstructural features could further enhance the thermoelectric performance of n-type SnSe films.^68^ Bismuth doping (6–8 wt %) enables stable n-type SnSe, though achieving optimal thermoelectric performance remains challenging. Recent advances in printed Bi-SnSe demonstrate its viability for device integration, with prototype generators showing significantly enhanced power output compared to p-type-only designs, highlighting Bi’s potential despite current *zT* limitations.^90^ Bi doping converts SnSe to n-type across synthesis methods, achieving carrier concentrations of 10^14^ - 10^18^ cm^−3^ with peak power factors of ∼5 μW cm^−1^K^−2^ (nanosheets) and Seebeck coefficients reaching −900 μVK^−1^ (bulk), while optimal doping reached *zT* ∼ 0.3–0.6.^67^^,^^69^^,^^81^

**Figure 12:**
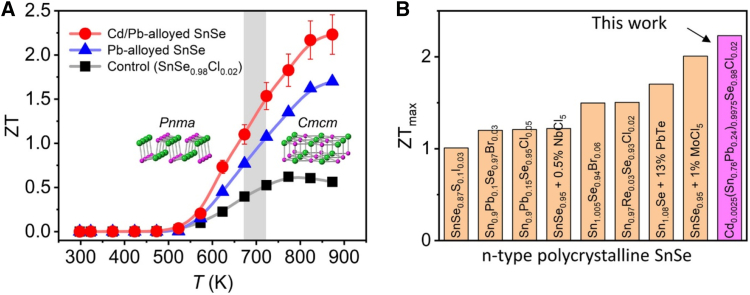
Thermoelectric performance of Cd/Pb-alloyed SnSe (A) Temperature-dependent *zT* values showing enhanced performance across the structural phase transition (673–723 K), compared to Pb-only and undoped samples. (B) Comparison of *zT*_max_ values with other leading n-type SnSe thermoelectric materials (reprinted from Byun et al.,^38^ Copyright 2021, with permission from Elsevier).

As a sole dopant, less commonly employed than Bi, lead has emerged as an alternative dopant for n-type SnSe, offering distinct advantages through its dual role as both electronic modifier and phonon scatterer. Significantly enhances electrical conductivity while reducing lattice thermal conductivity through mass and strain fluctuations, when combined with Ti co doping, the system achieves optimized carrier transport and phonon scattering, yielding *zT* ∼ 0.4 in Sn_0.74_Pb_0.20_Ti_0.06_Se.^92^ Pb doping in SnSe single crystals induces n-type conduction via Se vacancy formation and electron delocalization, achieving a power factor of 1.2 μWcm^−1^K^−1^ at room temperature.^82^ PbTe alloying (13%) in Br doped SnSe reduces the band gap by ∼ 30% and enhances crystal symmetry, boosting carrier concentration while maintaining high Seebeck coefficients (>150 μV/K). Combined with 8% excess Sn to passivate vacancies and form conductive grain boundary networks, this yields record *zT* ∼ 1.7 (793 K) and *zT*_ave_ ∼ 0.58 (300–793 K) a 40% improvement over undoped SnSe. The dual approach of band-gap engineering and defect control provides a blueprint for optimizing wide-band-gap thermoelectrics.^80^ Temperature-dependent measurements as shown in Figure 12A demonstrate that Cd/Pb-alloyed SnSe achieves a remarkable *zT* ∼ 2.23 at 873 K (parallel to SPS), maintaining stability across the Pnma to Cmcm phase transition (673–723 K) and showing a 31% improvement over Pb-only samples (*zT*∼1.70). The record-breaking nature of this performance is confirmed in Figure 12B, surpassing all known n-type polycrystalline materials including.^38^ Influence of key dopants on the thermoelectric performance of n-type polycrystalline SnSe is summarized in Table 3.

**Table 3:** A summary of doping influence on the thermoelectric performance of n-type polycrystalline SnSe

Dopant/strategy	Key composition	Peak zT (Temp.)	S (μV/K)	σ (S/cm)	PF (μW/cmK^2^)	Thermal conductivity (W/mK)	Reference
**Intrinsic**	SnSe	∼1.8 @ 816 K	−350 @ 800 K	–	10 @ 816 K	κ: 0.37 @ 873 K	Gainza et al.^87^
**Chlorides (Cl)**	SnSe + MoCl_5_	∼2.0 @ 798 K	−470 @ 827 K	41 @ 827 K	8.7 @ 827 K	κ_**lat**_: 0.26 @ 798 K	Chandra et al.^51^
	SnSe + WCl_6_	∼2.2 @ 773 K	–	39.2 @ 823 K	7.95 @ 773 K	κ_**L**_: 0.24 @773 K	Gong et al.^39^
	SnSe + SnCl_2_/Cu	∼1.55 @ 773 K	–	31 @ 773 K	7.25 @ 773 K	κ_**l**_: 0.29 @ 773 K	Li et al.^43^
	SnSe + ZnCl_2_	∼1.3 @ 873 K	−421 @ 873 K	32.4 @ 720 K	5.4 @ 873 K	κ_**l**_: 0.31 @ 773 K	Wu et al.^44^
**Bromides (Br)**	SnSe + Ge/Br	∼1.34 @ 773 K	−457 @ 773 K	31.8 @ 773 K	6.62 @ 773 K	κ: 0.38 @ 773 K	Nisar et al.^40^
	SnSe + Br	∼1.5 @ 783 K	–	29.5 @ 783 K	6.81 @ 783 K	κ_**lat**_: 0.32 @ 783 K	Shang et al.^85^
**Iodides (I)**	SnSe + I + MWCNT	∼1.0 @ 773 K	–	45.7 @ 773 K	5 @ 773 K	κ_**l**_: 0.34 @ 773 K	Mao et al.^70^
	SnSe + I	∼1.02 @ 723 K	–	25.9 @ 773 K	5.02 @ 723 K	κ_**latt**_: 0.30 @ 723 K	Abbas et al.^29^
	SnSe + I + WSe_2_	∼1.35 @ 790 K	∼471 @ 790 K	26.6 @ 790 K	5.9 @ 790 K	κ: 0.35 @ 790 K	Chen et al.^28^
**Complex alloys**	SnSe + Cd/Pb	∼2.23 @ 873 K	–	75.37 @ 873 K	8.8 @ 823 K	κ_**lat**_: 0.24 @ 873 K	Byun et al.^38^
	SnSe + TaSe_2_/Br	∼2.2 @ 823 K	−416 @ 823 K	–	7.1 @ 823 K	κ_**L**_: ∼0.18 @ 773 K	Taneja et al.^74^
	SnSe + PbTe	∼1.7 @ 793 K	–	60 @ 793 K	8.3 @ 793 K	κ_**lat**_: 0.31 @ 793 K	Su et al.^80^
**Rare earths**	SnSe + Ce + PbTe	∼1.3 @ 823 K	–	–	6.5 @ 823 K-	κ_**L**_: ∼0.39 @ 823 K	Li et al.^78^

## Conclusion and outlook

N-type SnSe has achieved remarkable progress through strategic doping (e.g., Cl/Br for band engineering, rare-earths for resonant levels) and microstructural control (e.g., texturing, nanocomposites), yet key challenges remain. Future efforts must address: (1) carrier concentration optimization to mitigate bipolar conduction at high temperatures, (2) scalable texturing techniques to exploit anisotropic transport, and (3) advanced characterization (e.g., *in situ* TEM) to resolve defect dynamics. Emerging strategies like high-entropy alloying (e.g., (Sn,Pb,Ge)Se) and heterointerface design (e.g., SnSe/MXene composites) offer avenues to further reduce lattice thermal conductivity while maintaining high power factors. Computational tools, particularly DFT coupled machine learning, could accelerate dopant discovery and phase stability prediction. By integrating these approaches, n-type SnSe may soon match p-type performance, enabling efficient, flexible, and cost-effective thermoelectric devices for energy harvesting and cooling applications.

## Acknowledgments

The work is financially supported by the 10.13039/100014717National Natural Science Foundation of China, China (project ID: U25A20581) and 10.13039/501100003392Fujian Provincial Natural Science Foundation Project (project ID: 2024J011540 and 2024CT018).

## Declaration of interests

The authors declare no competing interests.

## Author contributions

Conceptualization, A.A. and S.I.; formal analysis, C.A.A. and H.K.; investigation, J.N.A.H.; resources, T.M.; data curation, A.A., F.M., S.I., C.A.A., and H.B.A.H.; writing – original draft preparation, A.A.; writing – review and editing, A.A., H.K., S.I., Y.-X.C., and M.S.R.; visualization, S.I., C.A.A., and H.K.; supervision, Y.-X.C. and M.S.R.; project administration, A.A., Y.-X.C., and M.S.R.; funding acquisition, T.M.

## References

[1] Hu C., Xia K., Fu C., Zhao X., Zhu T. (2022). Carrier grain boundary scattering in thermoelectric materials. Energy Environ. Sci..

[2] Beretta D., Neophytou N., Hodges J.M., Kanatzidis M.G., Narducci D., Martin- Gonzalez M., Beekman M., Balke B., Cerretti G., Tremel W. (2019). Thermoelectrics: From history, a window to the future. Mater. Sci. Eng. R Rep..

[3] Chikina I., Goupil C., Sharapov S.G., Varlamov A.A. (2023). Thermoelectricity: from the iron arc of Alessandro Volta to radioisotope thermoelectric generators. Phys. Educ..

[4] Deng T., Qiu P., Yin T., Li Z., Yang J., Wei T., Shi X. (2024). High-Throughput Strategies in the Discovery of Thermoelectric Materials. Adv. Mater..

[5] Zhong Y., Tang J., Liu H., Chen Z., Lin L., Ren D., Liu B., Ang R. (2020). Optimized Strategies for Advancing n-Type PbTe Thermoelectrics: A Review. ACS Appl. Mater. Interfaces.

[6] Toral V., Gómez-Gijón S., Romero F.J., Morales D.P., Castillo E., Rodríguez N., Rojas S., Molina-Lopez F., Rivadeneyra A. (2024). Future Trends in Alternative Sustainable Materials for Low-Temperature Thermoelectric Applications. ACS Appl. Electron. Mater..

[7] Hong M., Chen Z.G. (2022). Chemistry in Advancing Thermoelectric GeTe Materials. Acc. Chem. Res..

[8] Jouhara H., Żabnieńska-Góra A., Khordehgah N., Doraghi Q., Ahmad L., Norman L., Axcell B., Wrobel L., Dai S. (2021). Thermoelectric generator (TEG) technologies and applications. Int. J. Thermofluids.

[9] Zhou S., Shi X.L., Li L., Liu Q., Hu B., Chen W., Zhang C., Liu Q., Chen Z.G. (2025). Advances and Outlooks for Carbon Nanotube-Based Thermoelectric Materials and Devices. Adv. Mater..

[10] Guan T., Gao J., Hua C., Tao Y., Ma Y., Liu J. (2025). Liquid Metal Enabled Thermoelectric Effects: Fundamental and Application. Adv. Funct. Mater..

[11] Chen Y.X., Nisar M., Qin W., Xu Z., Danish M.H., Li F., Liang G., Ge Z.H., Luo J., Zheng Z. (2025). Integration of Boron Nitride Into Tin-Enriched SnSe2 for a High-performance Thermoelectric Nanocomposite with Optimized Electrical Transport and Mechanical Properties. Adv. Funct. Mater..

[12] Wang D., Ding J., Ma Y., Xu C., Li Z., Zhang X., Zhao Y., Zhao Y., Di Y., Liu L. (2024). Multi-heterojunctioned plastics with high thermoelectric figure of merit. Nature.

[13] Abbas A., Nisar M., Zheng Z.H., Li F., Jabar B., Liang G., Fan P., Chen Y.X. (2022). Achieving High Thermoelectric Performance of Eco-Friendly SnTe-Based Materials by Selective Alloying and Defect Modulation. ACS Appl. Mater. Interfaces.

[14] Abbas A., Zhai J., Wang L., Li Z., Cao P., Wang C., Wang H. (2025). Grain size optimization of Mn doped SnTe materials starring to the improved thermoelectric and mechanical properties. Mater. Today Chem..

[15] Abbas A., Zhai J., Li Z., Cao P., Wang C., Wang H. (2025). Impact of sintering time and temperature on the thermoelectric properties of Sn0.97Mn0.06Te alloys. Ceram. Int..

[16] Li F., Zheng Z., Chang Y., Ruan M., Ge Z., Chen Y., Fan P. (2019). Synergetic Tuning of the Electrical and Thermal Transport Properties via Pb/Ag Dual Doping in BiCuSeO. ACS Appl. Mater. Interfaces.

[17] Mehta R.J., Zhang Y., Zhu H., Parker D.S., Belley M., Singh D.J., Ramprasad R., Borca-Tasciuc T., Ramanath G. (2012). Seebeck and figure of merit enhancement in nanostructured antimony telluride by antisite defect suppression through sulfur doping. Nano Lett..

[18] Jiang B., Wang W., Liu S., Wang Y., Wang C., Chen Y., Xie L., Huang M., He J. (2022). High figure-of-merit and power generation in high-entropy GeTe-based thermoelectrics. Science.

[19] Chen Y.X., Shi X.L., Zhang J.Z., Nisar M., Zha Z.Z., Zhong Z.N., Li F., Liang G.X., Luo J.T., Li M. (2024). Deviceization of high-performance and flexible Ag(2)Se films for electronic skin and servo rotation angle control. Nat. Commun..

[20] Pei J., Cai B., Zhuang H.-L., Li J.-F. (2020). Bi2Te3-based applied thermoelectric materials: research advances and new challenges. Natl. Sci. Rev..

[21] Witting I.T., Chasapis T.C., Ricci F., Peters M., Heinz N.A., Hautier G., Snyder G.J. (2019). The Thermoelectric Properties of Bismuth Telluride. Adv. Electron. Mater..

[22] Kumar A., Bano S., Govind B., Bhardwaj A., Bhatt K., Misra D.K. (2021). A Review on Fundamentals, Design and Optimization to High ZT of Thermoelectric Materials for Application to Thermoelectric Technology. J. Electron. Mater..

[23] Zhao L.-D., Lo S.-H., Zhang Y., Sun H., Tan G., Uher C., Wolverton C., Dravid V.P., Kanatzidis M.G. (2014). Ultralow thermal conductivity and high thermoelectric figure of merit in SnSe crystals. Nature.

[24] Chang C., Wu M., He D., Pei Y., Wu C.-F., Wu X., Yu H., Zhu F., Wang K., Chen Y. (2018). 3D charge and 2D phonon transports leading to high out-of-plane ZT in n-type SnSe crystals. Science.

[25] Qin B., Kanatzidis M.G., Zhao L.-D. (2024). The development and impact of tin selenide on thermoelectrics. Science.

[26] Zhou C., Lee Y.K., Yu Y., Byun S., Luo Z.-Z., Lee H., Ge B., Lee Y.-L., Chen X., Lee J.Y. (2021). Polycrystalline SnSe with a thermoelectric figure of merit greater than the single crystal. Nat. Mater..

[27] Jia B., Wu D., Xie L., Wang W., Yu T., Li S., Wang Y., Xu Y., Jiang B., Chen Z. (2024). Pseudo-nanostructure and trapped-hole release induce high thermoelectric performance in PbTe. Science.

[28] Chen Y.-X., Shi X.-L., Zheng Z.-H., Li F., Liu W.-D., Chen W.-Y., Li X.-R., Liang G.-X., Luo J.-T., Fan P., Chen Z.G. (2021). Two-dimensional WSe2/SnSe p-n junctions secure ultrahigh thermoelectric performance in n-type Pb/I Co-doped polycrystalline SnSe. Mater. Today Phys..

[29] Abbas A., Xu Z., Nisar M., Li D., Li F., Zheng Z., Liang G., Fan P., Chen Y.-X. (2022). Achieving weak anisotropy in N-type I-doped SnSe polycrystalline thermoelectric materials. J. Eur. Ceram. Soc..

[30] Lee Y.K., Luo Z., Cho S.P., Kanatzidis M.G., Chung I. (2019). Surface Oxide Removal for Polycrystalline SnSe Reveals Near-Single-Crystal Thermoelectric Performance. Joule.

[31] Duvjir G., Min T., Thi Ly T., Kim T., Duong A.-T., Cho S., Rhim S.H., Lee J., Kim J. (2017). Origin of p-type characteristics in a SnSe single crystal. Appl. Phys. Lett..

[32] Siddique S., Abbas G., Yaqoob M.M., Zhao J., Chen R., Larsson J.A., Cao Y., Chen Y., Zheng Z., Zhang D., Li F. (2025). Optimization of Thermoelectric Performance in p-Type SnSe Crystals Through Localized Lattice Distortions and Band Convergence. Adv. Sci..

[33] Nguyen V.Q., Trinh T.L., Chang C., Zhao L.-D., Nguyen T.H., Duong V.T., Duong A.T., Park J.H., Park S., Kim J., Cho S. (2022). Unidentified major p-type source in SnSe: Multivacancies. NPG Asia Mater..

[34] Gong Y., Dou W., Li Y., Ying P., Tang G. (2025). A Review of Polycrystalline SnSe Thermoelectric Materials: Progress and Prospects. Acta Metall. Sin. (English Letters).

[35] Shi X.L., Zou J., Chen Z.G. (2020). Advanced Thermoelectric Design: From Materials and Structures to Devices. Chem. Rev..

[36] Shi X.L., Tao X., Zou J., Chen Z.G. (2020). High-Performance Thermoelectric SnSe: Aqueous Synthesis, Innovations, and Challenges. Adv. Sci. (Weinh).

[37] Tan G., Zhao L.D., Kanatzidis M.G. (2016). Rationally Designing High-Performance Bulk Thermoelectric Materials. Chem. Rev..

[38] Byun S., Ge B., Song H., Cho S.-P., Hong M.S., Im J., Chung I. (2024). Simultaneously engineering electronic and phonon band structures for high-performance n-type polycrystalline SnSe. Joule.

[39] Gong Y., Dou W., Lu B., Zhang X., Zhu H., Ying P., Zhang Q., Liu Y., Li Y., Huang X. (2024). Divacancy and resonance level enables high thermoelectric performance in n-type SnSe polycrystals. Nat. Commun..

[40] Nisar M., Abbas A., Zhang J., Li F., Zheng Z., Liang G., Fan P., Chen Y.X. (2024). Anion/Cation Co-Doping to Improve the Thermoelectric Performance of Sn-Enriched n-Type SnSe Polycrystals with Suppressed Lattice Thermal Conductivity. Small.

[41] Ge Z.H., Qiu Y., Chen Y.X., Chong X., Feng J., Liu Z.K., He J. (2019). Multipoint Defect Synergy Realizing the Excellent Thermoelectric Performance of n-Type Polycrystalline SnSe via Re Doping. Adv. Funct. Mater..

[42] Cha J., Zhou C., Lee Y.K., Cho S.P., Chung I. (2019). High Thermoelectric Performance in n-Type Polycrystalline SnSe via Dual Incorporation of Cl and PbSe and Dense Nanostructures. ACS Appl. Mater. Interfaces.

[43] Li Z., Li W.J., Guo J., Wang Z.Y., Yang X., Zhu Y.K., Shi T.E., Zhang Y.X., Feng J., Ge Z.H. (2024). Heterogeneous Cu Doping Facilitates Excellent Thermoelectric and Mechanical Performance in n-Type SnSe Composites. ACS Appl. Mater. Interfaces.

[44] Wu X., Wu H., Liu J., Zheng S., Xiong Q., Zhang K., Zou H., Wang G., Han G., Wang G. (2025). Realizing High Thermoelectric Performance in ZnCl(2)-Doped N-Type Polycrystalline SnSe Through Band Engineering and Incorporating Multiple Defects. ACS Appl. Mater. Interfaces.

[45] Zhang Q., Chere E.K., Sun J., Cao F., Dahal K., Chen S., Chen G., Ren Z. (2015). Studies on Thermoelectric Properties of n-type Polycrystalline SnSe1-xSx by Iodine Doping. Adv. Energy Mater..

[46] Chang C., Tan Q., Pei Y., Xiao Y., Zhang X., Chen Y.-X., Zheng L., Gong S., Li J.-F., He J., Zhao L.D. (2016). Raising thermoelectric performance of n-type SnSe via Br doping and Pb alloying. RSC Adv..

[47] Shi H., Wen Y., Bai S., Chang C., Su L., Gao T., Liu S., Liu D., Qin B., Qin Y. (2025). Crystal symmetry modification enables high-ranged in-plane thermoelectric performance in n-type SnSe crystals. Nat. Commun..

[48] Yang X., Shi T.-E., Li W.-J., Ma X.-Y., Feng J., Ge Z.-H. (2023). Nanostructured n-Type Polycrystalline SnSe Materials for Thermoelectric Applications. ACS Appl. Nano Mater..

[49] Shi X.L., Zheng K., Liu W.D., Wang Y., Yang Y.Z., Chen Z.G., Zou J. (2018). Realizing High Thermoelectric Performance in n-Type Highly Distorted Sb-Doped SnSe Microplates via Tuning High Electron Concentration and Inducing Intensive Crystal Defects. Adv. Energy Mater..

[50] Liu H., Zhang S., Zhang Y., Zong S., Li W., Zhu C., Luo F., Wang J., Sun Z. (2022). Study on the Thermoelectric Properties of n-Type Polycrystalline SnSe by CeCl3 Doping. ACS Appl. Energy Mater..

[51] Chandra S., Bhat U., Dutta P., Bhardwaj A., Datta R., Biswas K. (2022). Modular Nanostructures Facilitate Low Thermal Conductivity and Ultra-High Thermoelectric Performance in n-Type SnSe. Adv. Mater..

[52] Yadav M., Singh V., Sharma S.K., Archana J., Navaneethan M., Pathak A., Bharti M., Singh A. (2024). Temperature driven n- to p-type conduction switching in SnSe and its mitigation through Zn doping with added advantage of Improved thermoelectric performance. Emergent. Mater..

[53] Byun J., An H., Hong J., Chun D.W., Moon J. (2021). Thermoelectric performance of n-type polycrystalline SnSe with surface depletion by pressureless sintering. Appl. Surf. Sci..

[54] Gu W.-H., Zhang Y.-X., Guo J., Cai J.-F., Zhu Y.-K., Zheng F., Jin L., Xu J., Feng J., Ge Z.-H. (2021). Realizing high thermoelectric performance in n-type SnSe polycrystals via (Pb, Br) co-doping and multi-nanoprecipitates synergy. J. Alloys Compd..

[55] Viet Chien N., Min Park H., Shin H., Yong Song J. (2023). Synthesis of n-type SnSe polycrystals with high and isotropic thermoelectric performance. J. Alloys Compd..

[56] Rundle J., Leoni S. (2022). Layered Tin Chalcogenides SnS and SnSe: Lattice Thermal Conductivity Benchmarks and Thermoelectric Figure of Merit. J. Phys. Chem. C Nanomater. Interfaces.

[57] Yang S., Liu Y., Wu M., Zhao L.-D., Lin Z., Cheng H.-c., Wang Y., Jiang C., Wei S.-H., Huang L. (2018). Highly-anisotropic optical and electrical properties in layered SnSe. Nano Res..

[58] Wu P., Ishikawa Y., Hagihala M., Lee S., Peng K., Wang G., Torii S., Kamiyama T. (2018). Crystal structure of high-performance thermoelectric materials by high resolution neutron powder diffraction. Phys. B Condens. Matter.

[59] Yang J., Zhang Y., Ge Y., Tang S., Li J., Zhang H., Shi X., Wang Z., Tian X. (2024). Interlayer Engineering of Layered Materials for Efficient Ion Separation and Storage. Adv. Mater..

[60] Zhang L., Wang J., Sun Q., Qin P., Cheng Z., Ge Z., Li Z., Dou S. (2017). Three-Stage Inter-Orthorhombic Evolution and High Thermoelectric Performance in Ag-Doped Nanolaminar SnSe Polycrystals. Adv. Energy Mater..

[61] Wan D., Bai S., Li X., Ai P., Guo W., Zhang J., Tang S. (2024). Anharmonicity and weak bonding-driven extraordinary thermoelectric performance in wrinkled SnSe monolayer with low lattice thermal conductivity. Ceram. Int..

[62] Kutorasinski K., Wiendlocha B., Kaprzyk S., Tobola J. (2015). Electronic structure and thermoelectric properties of $n$- and $p$-type SnSe from first-principles calculations. Phys. Rev. B.

[63] Yang J., Zhang G., Yang G., Wang C., Wang Y.X. (2015). Outstanding thermoelectric performances for both p- and n-type SnSe from first-principles study. J. Alloys Compd..

[64] Sun J., Guo D., Zhang H., Xu Z., Li C., Li K., Shao B., Chen D., Ma Y. (2022). Electron mean-free-path filtering in n-type SnSe for improved thermoelectric performance at room temperature. J. Alloys Compd..

[65] Guo T., Guo D. (2025). The optimal grain boundary enhances the thermoelectric performance of SnSe by 40–50%. J. Alloys Compd..

[66] Chaves A.S., Larson D.T., Kaxiras E., Antonelli A. (2021). Microscopic origin of the high thermoelectric figure of merit of n-doped SnSe. Phys. Rev. B.

[67] Li X., Chen C., Xue W., Li S., Cao F., Chen Y., He J., Sui J., Liu X., Wang Y., Zhang Q. (2018). N-type Bi-doped SnSe Thermoelectric Nanomaterials Synthesized by a Facile Solution Method. Inorg. Chem..

[68] Horide T., Nakamura K., Hirayama Y., Morishita K., Ishimaru M., Matsumoto K. (2021). Thermoelectric Property of n-Type Bismuth-Doped SnSe Film: Influence of Characteristic Film Defect. ACS Appl. Energy Mater..

[69] Chandra S., Banik A., Biswas K. (2018). n-Type Ultrathin Few-Layer Nanosheets of Bi-Doped SnSe: Synthesis and Thermoelectric Properties. ACS Energy Lett..

[70] Mao X.-Y., Shi X.-L., Zhai L.-C., Liu W.-D., Chen Y.-X., Gao H., Li M., Wang D.-Z., Wu H., Zheng Z.-H. (2022). High thermoelectric and mechanical performance in the n-type polycrystalline SnSe incorporated with multi-walled carbon nanotubes. J. Mater. Sci. Technol..

[71] Li S., Wang Y., Chen C., Li X., Xue W., Wang X., Zhang Z., Cao F., Sui J., Liu X., Zhang Q. (2018). Heavy Doping by Bromine to Improve the Thermoelectric Properties of n-type Polycrystalline SnSe. Adv. Sci. (Weinh.).

[72] Zhang S., Zhu C., He X., Wang J., Luo F., Wang J., Liu H., Sun Z. (2022). Enhanced thermoelectric performance of n-type polycrystalline SnSe via NdCl3 doping. J. Alloys Compd..

[73] Shi T.-E., Yang X., Li W.-J., Li Z., Wang Z.-Y., Zhang Y.-X., Feng J., Ge Z.-H. (2025). Multiscale-defects simultaneous optimization of thermoelectric performance in the n-type polycrystalline SnSe via (Zr, Cl) Co-doping. J. Alloys Compd..

[74] Taneja V., Goyal N., Das S., Chandra S., Dutta P., Ravishankar N., Biswas K. (2024). Nanostructured Ferecrystal Intergrowths with TaSe(2) Unveiled High Thermoelectric Performance in n-Type SnSe. J. Am. Chem. Soc..

[75] Shen T., Li K.Y., Chen Z.J., Wu H.F., Si J.X. (2019). Enhanced Thermoelectric Performance of n-Type Polycrystalline SnSe via MoCl5 Doping. J. Electron. Mater..

[76] Li Z., Wang Y., Liu D., Hong T., Qin B., Gao X., Zhao L.-D. (2025). Attempts to realize promising thermoelectric performance in n-type polycrystalline SnSe with a cubic structure. J. Mater. Chem. A Mater..

[77] Cai J., Zhang Y., Yin Y., Tan X., Duan S., Liu G.-Q., Hu H., Xiao Y., Ge Z., Jiang J. (2020). Investigating the thermoelectric performance of n-type SnSe: the synergistic effect of NbCl5 doping and dislocation engineering. J. Mater. Chem. C Mater..

[78] Li S., Yin L., Liu Y., Wang X., Chen C., Zhang Q. (2023). Rare earth element Ce enables high thermoelectric performance in n-type SnSe polycrystals. J. Mater. Sci. Technol..

[79] Yang X., Gu W.-H., Li W.-J., Zhang Y.-X., Feng J., Ge Z.-H. (2023). Improved thermoelectric properties of n-type polycrystalline SnSe via carrier concentration optimization. J. Phys. Chem. Solid..

[80] Su L., Hong T., Wang D., Wang S., Qin B., Zhang M., Gao X., Chang C., Zhao L.-D. (2021). Realizing high doping efficiency and thermoelectric performance in n-type SnSe polycrystals via bandgap engineering and vacancy compensation. Mater. Today Phys..

[81] Nguyen V.Q., Nguyen T.H., Duong V.T., Lee J.E., Park S.D., Song J.Y., Park H.M., Duong A.T., Cho S. (2018). Thermoelectric Properties of Hot-Pressed Bi-Doped n-Type Polycrystalline SnSe. Nanoscale Res. Lett..

[82] Tang Y., Shen L., Chen Z., Sun L., Liu W., Liu J., Deng S. (2019). The N-type Pb-doped single crystal SnSe thermoelectric material synthesized by a Sn-flux method. Phys. B Condens. Matter.

[83] Li D., Tan X., Xu J., Liu G., Jin M., Shao H., Huang H., Zhang J., Jiang J. (2017). Enhanced thermoelectric performance in n-type polycrystalline SnSe by PbBr2 doping. RSC Adv..

[84] Li S., Zhang F., Chen C., Li X., Cao F., Sui J., Liu X., Ren Z., Zhang Q. (2020). Enhanced thermoelectric performance in polycrystalline N-type Pr-doped SnSe by hot forging. Acta Mater..

[85] Shang P.P., Dong J., Pei J., Sun F.H., Pan Y., Tang H., Zhang B.P., Zhao L.D., Li J.F. (2019). Highly Textured N-Type SnSe Polycrystals with Enhanced Thermoelectric Performance. Research (Wash. D C).

[86] Gainza J., Serrano-Sánchez F., Gharsallah M., Carrascoso F., Bermúdez J., Dura O.J., Mompean F.J., Biskup N., Meléndez J.J., Martínez J.L. (2019). Evidence of nanostructuring and reduced thermal conductivity in n-type Sb-alloyed SnSe thermoelectric polycrystals. J. Appl. Phys..

[87] Gainza J., Serrano-Sánchez F., Rodrigues J.E.F.S., Huttel Y., Dura O.J., Koza M.M., Fernández-Díaz M.T., Meléndez J.J., Márkus B.G., Simon F. (2020). High-Performance n-type SnSe Thermoelectric Polycrystal Prepared by Arc-Melting. Cell Rep. Phys. Sci..

[88] Mao L., Yin Y., Zhang Q., Liu G.-Q., Wang H., Guo Z., Hu H., Xiao Y., Tan X., Jiang J. (2020). Fermi-surface dynamics and high thermoelectric performance along the out-of-plane direction in n-type SnSe crystals. Energy Environ. Sci..

[89] Li S., Hou S., Liu H., Yin L., Bao X., Li J., Wang X., Ye X., Zhang Q. (2023). Realizing high thermoelectric performance in CeCl3-doped n-type SnSe polycrystals. Ceram. Int..

[90] Burton M.R., Howells G., Mehraban S., McGettrick J.D., Lavery N., Carnie M.J. (2023). Fully 3D Printed Tin Selenide (SnSe) Thermoelectric Generators with Alternating n-Type and p-Type Legs. ACS Appl. Energy Mater..

[91] Su L., Shi H., Wang S., Wang D., Qin B., Wang Y., Chang C., Zhao L.D. (2023). Enhancing Carrier Mobility and Seebeck Coefficient by Modifying Scattering Factor. Adv. Energy Mater..

[92] Li F., Wang W., Qiu X., Zheng Z., Fan P., Luo J., Li B. (2017). Optimization of thermoelectric properties of n-type Ti, Pb co-doped SnSe. Inorg. Chem. Front..

